# Waste-Derived Zeolite-P (GIS): Synthesis, Polymorphism, Characterization, and Adsorption Applications for Water Purification and Selective Gas Separation

**DOI:** 10.3390/molecules31203720

**Published:** 2026-10-16

**Authors:** Rafik Abdelkrim Boudia, Stefano Bellucci, Mehdi Adjdir

**Affiliations:** 1 Laboratory of Applied Organic Synthesis, Faculty of Exact and Applied Sciences, University Oran1 Ahmed Ben Bella, BP 1524 El M’Naouer, Oran 31000, Algeria; 2 Laboratory of Elaboration and Spectroscopic Characterizations of Structural and Electronic Properties Materials, Faculty of Technology, University of Saida Tahar Moulay, P.B. 138 Ennasr, Saida 20000, Algeria; 3 Qi S.r.l., Via Monte d’Oro, 2/A, 00071 Pomezia, Italy; 4 Research Direction, Universidad ECOTEC, Km 13.5 Samborondón, Samborondón 092302, Ecuador; 5 National Institute of Materials Physics (NIMP), 405A Atomistilor Street, 077125 Magurele, Romania

**Keywords:** GIS polymorphs, waste-derived zeolite-P, microporosity, hierarchical porosity, phase purity, water purification, regeneration, selective gas separation

## Abstract

Zeolite-P is a synthetic member of the gismondine (GIS) framework family and is intrinsically microporous, with intersecting eight-membered-ring channels. Mesoporosity reported for some Zeolite-P samples is therefore a secondary textural feature arising from interparticle voids, crystal aggregation, defects, or deliberately hierarchical architectures rather than from the GIS framework itself. This critical review focuses specifically on waste-derived GIS/Zeolite-P and integrates polymorph selection (Na-P1 and Na-P2), phase-purity assessment, precursor-to-property relationships, shaping, regeneration, and adsorption under increasingly realistic conditions. Structural and compositional evidence from X-ray diffraction (XRD), scanning electron microscopy (SEM), Fourier-transform infrared spectroscopy (FT-IR), X-ray fluorescence (XRF), inductively coupled plasma optical emission spectroscopy (ICP-OES), solid-state ^29^Si and ^27^Al magic-angle-spinning nuclear magnetic resonance (MAS NMR), X-ray photoelectron spectroscopy (XPS), and Brunauer–Emmett–Teller (BET) analysis is evaluated in terms of what each technique can establish specifically for GIS materials. Waste-derived synthesis routes—including hydrothermal, alkali-fusion-assisted, microwave-assisted, ultrasound-assisted, sol–gel, and solvent-free methods—are compared without assuming that a waste precursor or a shorter synthesis is intrinsically lower-impact. Particular attention is paid to water purification, ion exchange, dehydration, and selective gas adsorption, while distinguishing single-solute batch tests from multicomponent, real-water, regeneration, and breakthrough evidence. The review also reconciles computational predictions with experiments: idealized pure-silica GIS models can overpredict CO_2_ uptake relative to realistic aluminosilicate Na-P1/Na-P2, for which extra-framework cations, hydration, and restricted diffusion strongly affect accessibility. The resulting analysis identifies phase purity, matrix effects, regeneration, leaching safety, shaping, and process-level techno-economic/life-cycle metrics as the key barriers to translation of waste-derived Zeolite-P from laboratory studies to deployable adsorbents.

## 1. Introduction

Separation processes account for a substantial fraction of industrial energy use and cost. Conventional operations include distillation, evaporation, absorption/stripping, extraction, crystallization, and membrane separations; adsorption is attractive when a selective solid can reduce the thermal duty required for purification [1,2,3,4]. In this context, zeolites are important because their crystalline pore networks combine molecular-size discrimination with ion-exchange chemistry and regenerability.

Among zeolitic adsorbents, the gismondine (GIS) framework is a small-pore aluminosilicate family. In this review, “GIS” denotes the framework topology, “Zeolite-P” denotes synthetic GIS-type P zeolites, and Na-P1 (P1) and Na-P2 (P2) denote the two principal sodium polymorphs discussed throughout. For comparison, framework codes such as faujasite (FAU), Linde Type A (LTA), MFI, and beta (BEA) are used according to International Zeolite Association nomenclature. Zeolite-P has the general composition M_2_/nO·Al_2_O_3_·1.8–5SiO_2_·5H_2_O, where M is an exchangeable cation [5]. P1 is commonly more Al-rich, whereas P2 is relatively silica-richer and thermally more stable [6,7].

GIS-type materials have been studied as ion exchangers and adsorbents for radionuclides, Pb^2+^, Zn^2+^, Ni^2+^, Mn^2+^, Cd^2+^, Sr^2+^, Ba^2+^, Cr^3+^/Cr^6+^, Hg^2+^, NH_4_^+^, phosphate, organic dyes, water vapor, and light gases [8,9,10,11,12]. Catalytic uses also exist, but they are substantially less mature than adsorption and ion exchange; accordingly, catalysis is treated here as a secondary application rather than as a co-equal focus of the review.

Zeolite-P can be prepared by conventional hydrothermal crystallization and by alkali-fusion-assisted, sol–gel, microwave-assisted, ultrasound-assisted, interzeolite, and solvent-free routes. Phase outcome depends on alkalinity, Si/Al and Si/OH ratios, water content, temperature, aging, crystallization time, and the identity and concentration of charge-balancing cations [7,13,14]. These variables are especially important for waste-derived feeds because precursor mineralogy and the fraction of amorphous Si/Al determine dissolution and nucleation behavior.

Industrial and agro-industrial residues—including coal fly ash, gasification slag, rice-husk ash, sugarcane bagasse ash, paper sludge ash, waste glass, and clay-rich fractions—can provide Si and Al for GIS synthesis. Their use can reduce demand for virgin precursors and divert waste from disposal, but “waste-derived” should not be used as a synonym for “low-impact”: transport, drying, milling, washing, acid/alkali pretreatment, high-temperature fusion, water consumption, and treatment of alkaline effluents can dominate the environmental and economic balance. Residual hazardous elements from fly ash or glass-derived feeds also require leaching and product-stability assessment before environmental deployment [15].

Recent reviews have surveyed zeolites from industrial waste broadly [15] or zeolite adsorption processes across many framework types [16]. The specific gap addressed here is narrower: a GIS/Zeolite-P-centered comparison that links P1/P2 polymorphism and phase purity to precursor chemistry, textural characterization, adsorption performance, regeneration, shaping, and computational/experimental discrepancies. This focus is important because results reported simply as “Zeolite-P” are not directly comparable when the products differ in polymorph, residual secondary phases, cation form, crystal size, or hierarchical porosity.

This article is a critical narrative review rather than a systematic review and therefore does not claim exhaustive coverage. The literature is organized around evidence needed for material and process translation: (i) unambiguous terminology and phase identification; (ii) GIS-specific interpretation of characterization data; (iii) synthesis from pure and waste-derived precursors; (iv) cost and environmental caveats; (v) powder-to-shaped adsorbent trade-offs; (vi) adsorption under model, multicomponent, and real-water conditions; (vii) regeneration and reuse; and (viii) reconciliation of molecular simulation with equilibrium and breakthrough experiments.

This review is restricted to GIS-type Zeolite-P (Na-P1/Na-P2) obtained from waste-derived precursors and to three classes of processes: ion exchange/adsorption in water treatment, selective gas adsorption, and, as a secondary and less mature field, catalysis. For each, we ask which physicochemical properties govern performance (framework type, crystallinity, Si/Al ratio, porosity, purity, exchange capacity, stability and acid–base sites) and whether the waste origin of the material acts directly or only through those properties. To avoid repetition, route-specific descriptions have been condensed and the discussion is organized around comparative mechanisms and process-relevant properties.

### Zeolites: Structural Context for GIS-Type Zeolite-P

**Figure 1:**
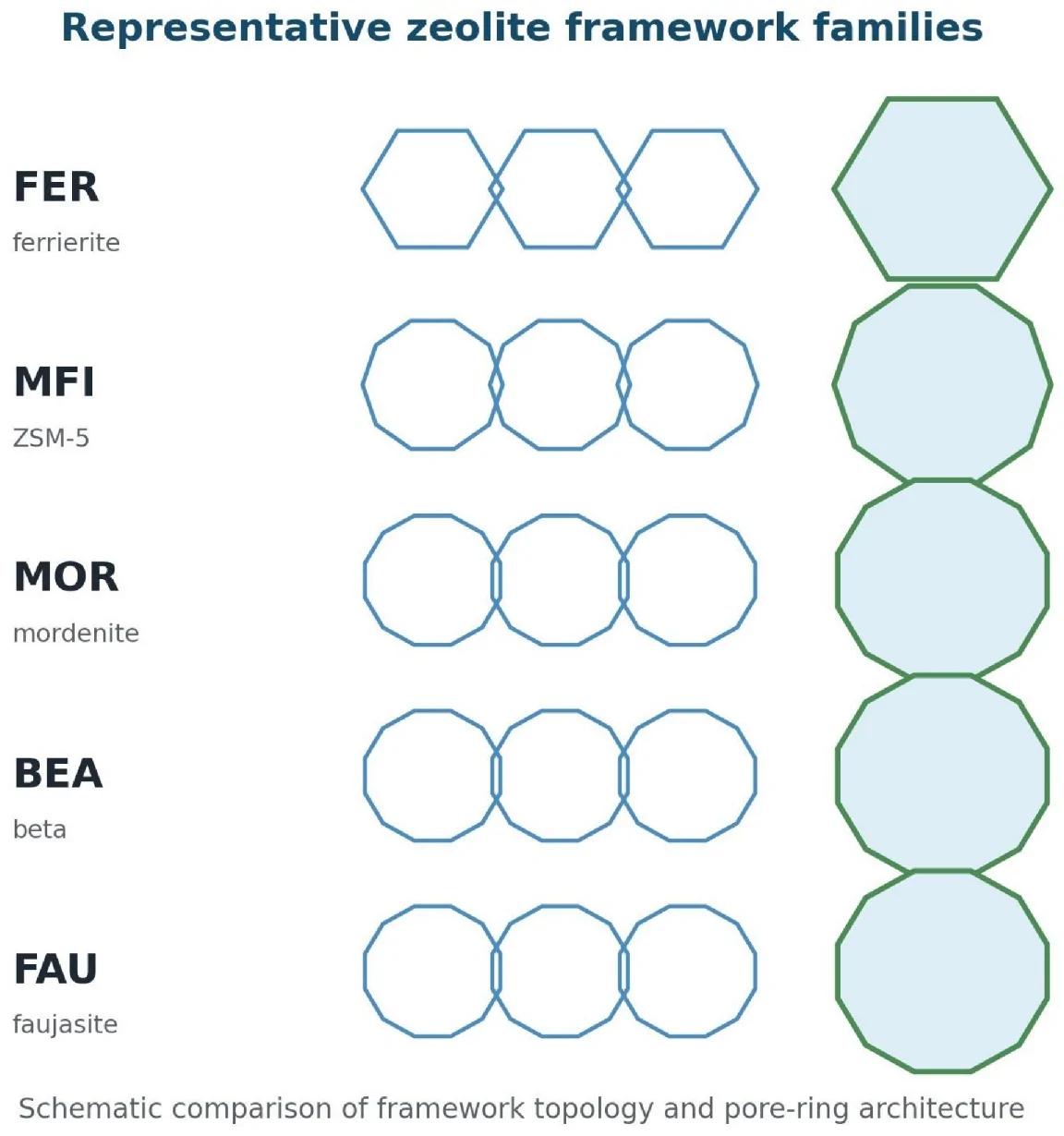
Original schematic comparison of representative zeolite framework families (FER, MFI, MOR, BEA, and FAU), based on standard framework nomenclature and structural descriptions in the International Zeolite Association (IZA) Structure Database and cited literature; no source artwork is reproduced.

Zeolites are crystalline microporous framework materials constructed from corner-sharing TO_4_ tetrahedra (T commonly Si and Al). Framework topology, Si/Al ratio, extra-framework cations, and crystal size jointly determine pore accessibility, ion-exchange capacity, acidity/basicity, diffusion, and stability [17,18]. For the present review, this general context is retained only to position GIS relative to larger-pore frameworks such as FAU, LTA, MFI, MOR, and BEA (Figure 1). The distinguishing feature of Zeolite-P is not an exceptionally high surface area, but a small, charge-bearing GIS pore system combined with exchangeable cations and the possibility of synthesis from diverse Si/Al-rich residues.

Relative to metal–organic frameworks and carbonaceous sorbents, Zeolite-P offers a crystalline, ion-exchange-active framework with good aqueous chemical stability and the possibility of regeneration. Its principal limitations are small-pore diffusion constraints, cation-dependent accessibility, and the need to shape powders for realistic fixed-bed use. These trade-offs motivate the GIS-specific comparisons developed below rather than a general survey of zeolite chemistry.

## 2. Gismondine-Type Zeolite (GIS-Zeolite): Zeolite-P

### 2.1. A Short History of Zeolite-P

Zeolite-P was established as a synthetic GIS-type material through structural and ion-exchange studies from the late 1960s onward. Early work by Ciric, Baerlocher and Meier, and Barrer and Munday established P-type synthesis, GIS topology, and reversible cation exchange [5,19,20,21]. Subsequent crystallographic work resolved Na-P1/Na-P2 variants and demonstrated the framework flexibility that underpins their different stability and sorption behavior [6,22].

The later literature shifted toward low-cost precursors, radionuclide and heavy-metal removal, detergent builders, and controlled P1/P2 crystallization. These developments are relevant here because they connect phase identity and cation exchange to the current emphasis on waste valorization and selective adsorption; a full chronological history is therefore not repeated.

### 2.2. GIS-Type-Zeolite: Zeolite-P

Gismondine (GIS)-type zeolites are intrinsically microporous aluminosilicates. Synthetic Zeolite-P contains intersecting eight-membered-ring channels reported at approximately 0.31 × 0.44 nm and 0.26 × 0.49 nm, with an effective micropore size near 0.29 nm in Na-P1 [23]. Two principal sodium polymorphs are considered here: Na-P1 (P1), commonly more Al-rich and often described as cubic/pseudo-cubic, and Na-P2 (P2), a silica-richer tetragonal polymorph [6,7]. These labels describe polymorphs of the same GIS framework, not separate framework types.

The negatively charged aluminosilicate framework is compensated by extra-framework cations, and the number, size, hydration state, and location of these cations strongly affect ion exchange and molecular access. Consequently, the same nominal GIS topology can show very different adsorption behavior after changes in Si/Al ratio or cation form. The literature therefore needs to distinguish framework topology, polymorph identity, cation form, and phase purity when comparing adsorption data.

**Figure 2:**
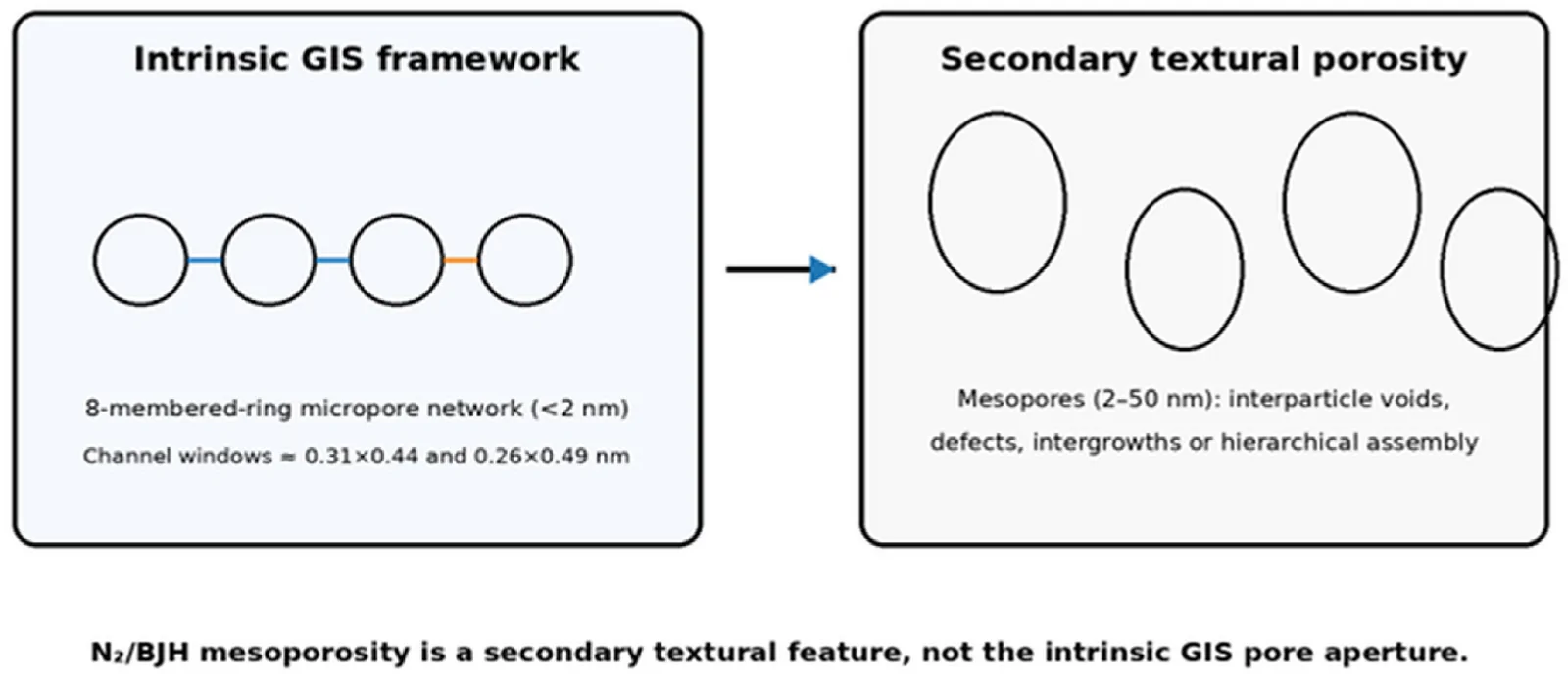
Schematic distinction between the intrinsic 8-membered-ring micropore network of GIS-type Zeolite-P and secondary textural mesoporosity arising from interparticle voids, intergrowths, defects, or hierarchical assembly. The channel-window values shown are framework-scale descriptors; N_2_/BJH mesoporosity should not be interpreted as the intrinsic GIS aperture.

A second terminology issue concerns porosity. The GIS framework itself is microporous; mesoporosity reported from N_2_ sorption, microscopy, or BJH analysis is secondary textural porosity generated by intercrystalline voids, aggregated nanocrystals, defects, or intentionally hierarchical structures. Such mesopores can shorten diffusion paths and improve mass transfer, but they should not be presented as the intrinsic pore aperture of GIS (Figure 2).

## 3. GIS Structure and Characterization

### 3.1. Topological Structure of GIS and Its Related Structures

**Figure 3:**
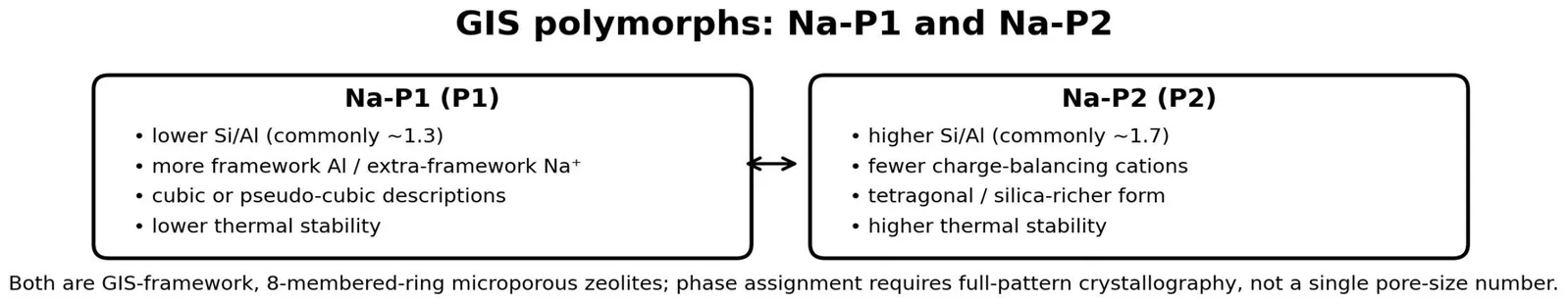
Comparison of Na-P1 (P1) and Na-P2 (P2) as polymorphs of the same GIS framework. The figure emphasizes the typical differences in Si/Al ratio, extra-framework cation population, symmetry descriptors, and relative thermal stability rather than assigning the larger crystallographic geometric dimensions as free pore apertures.

P1 and P2 share the GIS topology but differ in composition, symmetry, cation population, and thermal stability. For adsorption and molecular sieving, the relevant framework-scale descriptors are the small 8-membered-ring channels (~0.31 × 0.44 and 0.26 × 0.49 nm for Na-P-type GIS) rather than larger crystallographic geometric distances that do not represent the free pore aperture. Accordingly, the previously used ~7 Å dimensions are not treated here as accessible pore diameters. P1/P2 identification should instead rely on full crystallographic patterns, composition, and structural refinement [7,23]. (Figure 3).

### 3.2. Characterization of the GIS Zeolite

#### 3.2.1. X-Ray Diffraction (XRD) Analysis of Zeolite-P

**Figure 4:**
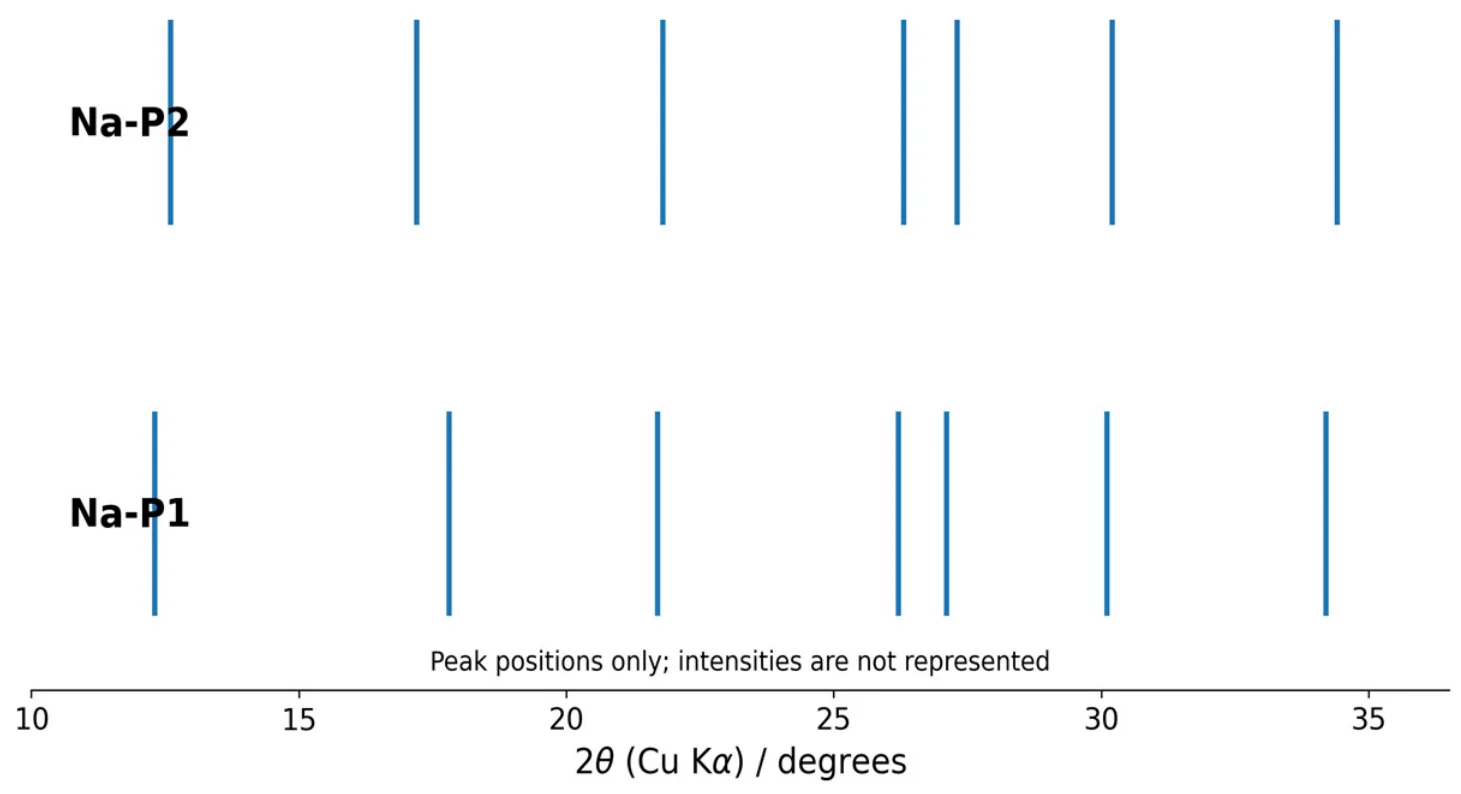
Schematic comparison of representative XRD reflection positions reported for Na-P1 and Na-P2. The small differences in some 2θ values are not, by themselves, a reliable phase-purity criterion; full-pattern comparison with crystallographic references is required [7,14,24].

For GIS-type Zeolite-P, X-ray diffraction (XRD) is primarily a phase-identification and phase-purity tool. Because several P1/P2 reflections occur at similar 2θ values, a single peak or a small peak shift is not sufficient to distinguish the polymorphs reliably. Phase assignment should use the full diffraction pattern and, where quantitative purity is claimed, whole-pattern fitting/Rietveld analysis or another explicit quantitative method. Samples containing sodalite, faujasite, analcime, residual quartz/mullite, or both P1 and P2 are treated in this review as mixed-phase materials rather than as phase-pure Zeolite-P. (Figure 4). (Table 1).

P1/P2 phase assignment should be based on the complete pattern and, when purity is quantified, full-pattern analysis.

Oleksiak et al. [7] demonstrated that synthesis composition can shift the product between P1, P2, and mixed-phase regions. Likewise, Boudia et al. [14] observed composition-dependent P1/P2 formation from clay-derived feeds. These studies illustrate why XRD claims should explicitly state whether “Zeolite-P” is phase-pure P1, phase-pure P2, a P1/P2 mixture, or a GIS-containing multiphase product.

Throughout the tables in this review, phase-purity statements are therefore retained only when supported by the cited study; where quantitative purity was not established, the product is described according to the phase(s) reported by the original authors rather than being reclassified as pure Zeolite-P.

#### 3.2.2. Scanning Electron Microscopy (SEM) Analysis

Scanning electron microscopy (SEM) is most useful here for linking crystal size, aggregation, and morphology to synthesis conditions and mass-transfer length. In clay-derived systems, Boudia et al. [14] reported morphology changes associated with P1/P2 formation, while Oleksiak et al. [7] related P1/P2 synthesis conditions to distinct crystal habits. SEM alone cannot establish phase purity, but it is a valuable complement to XRD when interpreting crystal growth and secondary textural porosity.

**Figure 5:**
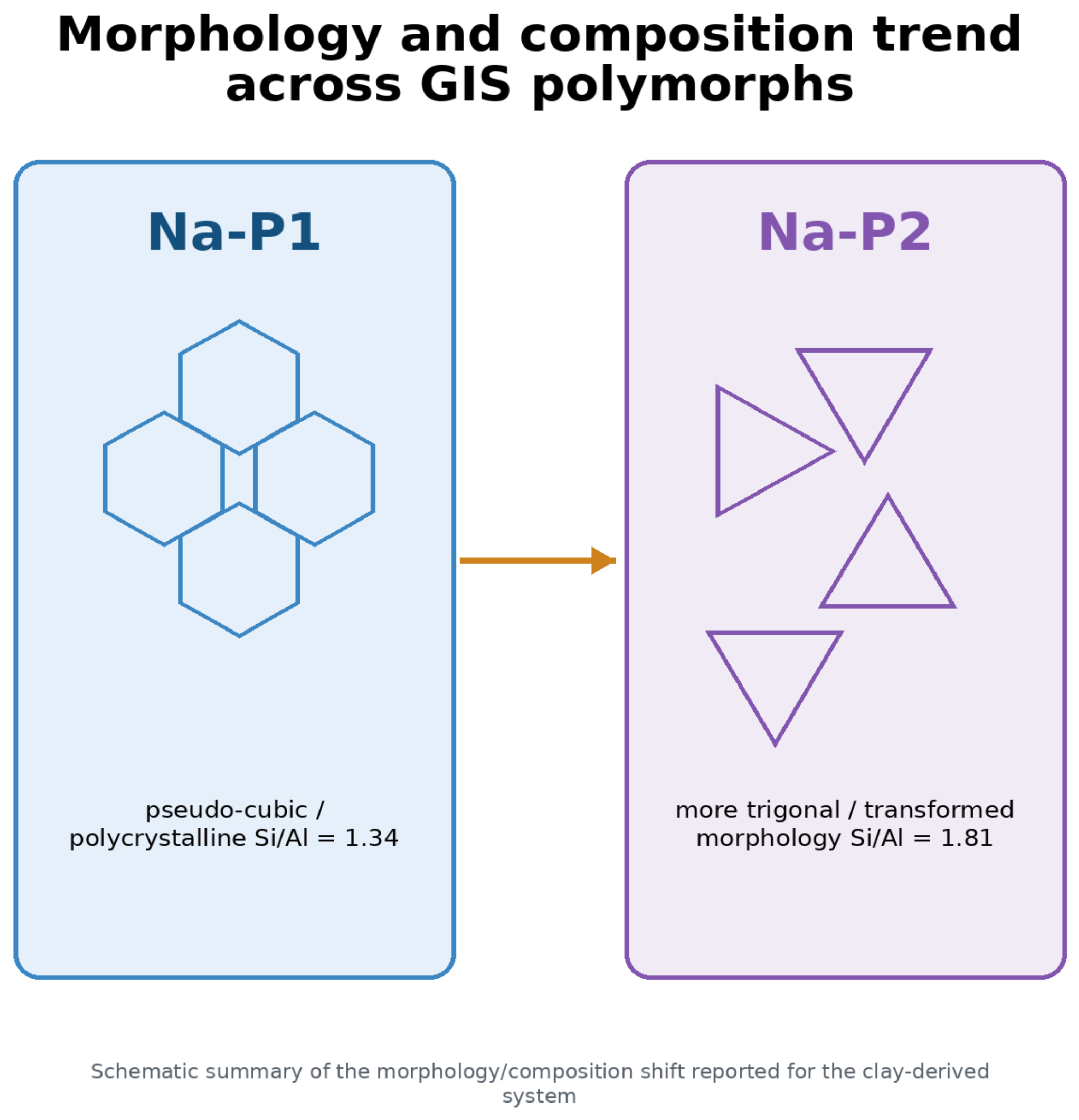
Original schematic summary of the morphology/composition trend reported for clay-derived GIS polymorphs: Na-P1 (representative Si/Al = 1.34) and Na-P2 (representative Si/Al = 1.81) [14]. The schematic does not reproduce published SEM or EDS artwork.

Crystal size matters because intracrystalline diffusion time scales approximately with L^2^/D. Smaller crystals and hierarchical interparticle porosity can therefore reduce diffusion limitations without changing the intrinsic GIS micropore topology. Conversely, large aggregates and binder-filled interparticle space can slow uptake even when the crystallographic phase is unchanged [16,23]. (Figure 5). (Table 2).

#### 3.2.3. FT-IR Analysis

**Figure 6:**
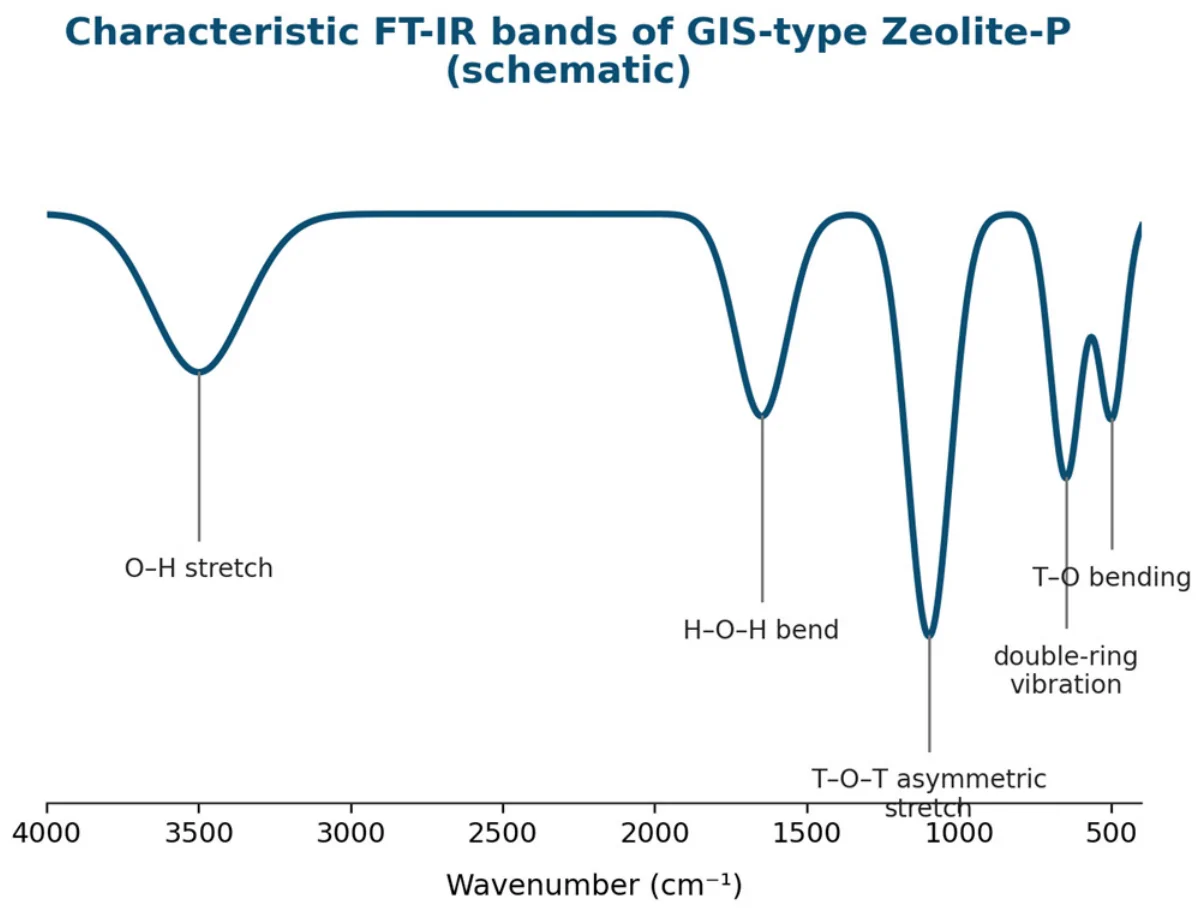
Original schematic FT-IR band-assignment diagram for GIS-type Zeolite-P, summarizing the characteristic O–H stretching, H–O–H bending, T–O–T asymmetric stretching, double-ring vibration, and T–O bending regions discussed in Section 3.2.3 [25].

Fourier-transform infrared (FT-IR) spectroscopy is used in GIS studies mainly to confirm aluminosilicate framework vibrations, hydration, and changes in surface hydroxyl environments. Bands in the ~1300–400 cm^−1^ region report T–O/T–O–T framework vibrations, while O–H/H–O–H bands report hydration and hydroxyl populations. FT-IR is supportive rather than definitive for P1/P2 phase assignment and should be interpreted together with XRD and compositional data [25]. (Figure 6). (Table 3).

#### 3.2.4. X-Ray Fluorescence (XRF) Spectrometry

X-ray fluorescence (XRF) is most relevant to waste-derived GIS as a bulk compositional screen for precursor SiO_2_/Al_2_O_3_ ratios and potentially problematic major/minor elements. The measured Si/Al ratio constrains synthesis design but does not by itself prove framework incorporation; pairing XRF with XRD and local probes is therefore essential. XRF is also useful for comparing feed and product compositions when evaluating impurity retention [26]. (Table 4).

#### 3.2.5. Inductively Coupled Plasma Optical Emission Spectrometer (ICP-OES)

Inductively coupled plasma optical emission spectroscopy (ICP-OES) complements XRF by quantifying dissolved species in leachates, synthesis liquors, wash waters, and digestion solutions. In GIS synthesis it can be used to follow Si/Al dissolution, residual Na and other cations, and potential hazardous-element release. This is particularly important for waste-derived products because bulk conversion to a zeolite does not guarantee that trace metals are immobilized under use or regeneration conditions [14]. (Table 5).

#### 3.2.6. Brunauer-Emmett-Teller Surface Area Analysis

Brunauer–Emmett–Teller (BET) analysis of N_2_ adsorption is frequently reported for Zeolite-P, but the resulting surface area must be interpreted cautiously for small-pore GIS. Restricted access of N_2_ or Ar to cation-blocked micropores can yield low apparent BET areas even for crystalline material, while type-IV/H3-type hysteresis can reflect secondary meso-/macroporosity between aggregated crystallites. BET/BJH data therefore characterize accessible texture under the probe conditions rather than defining the crystallographic GIS aperture [23,27].

**Figure 7:**
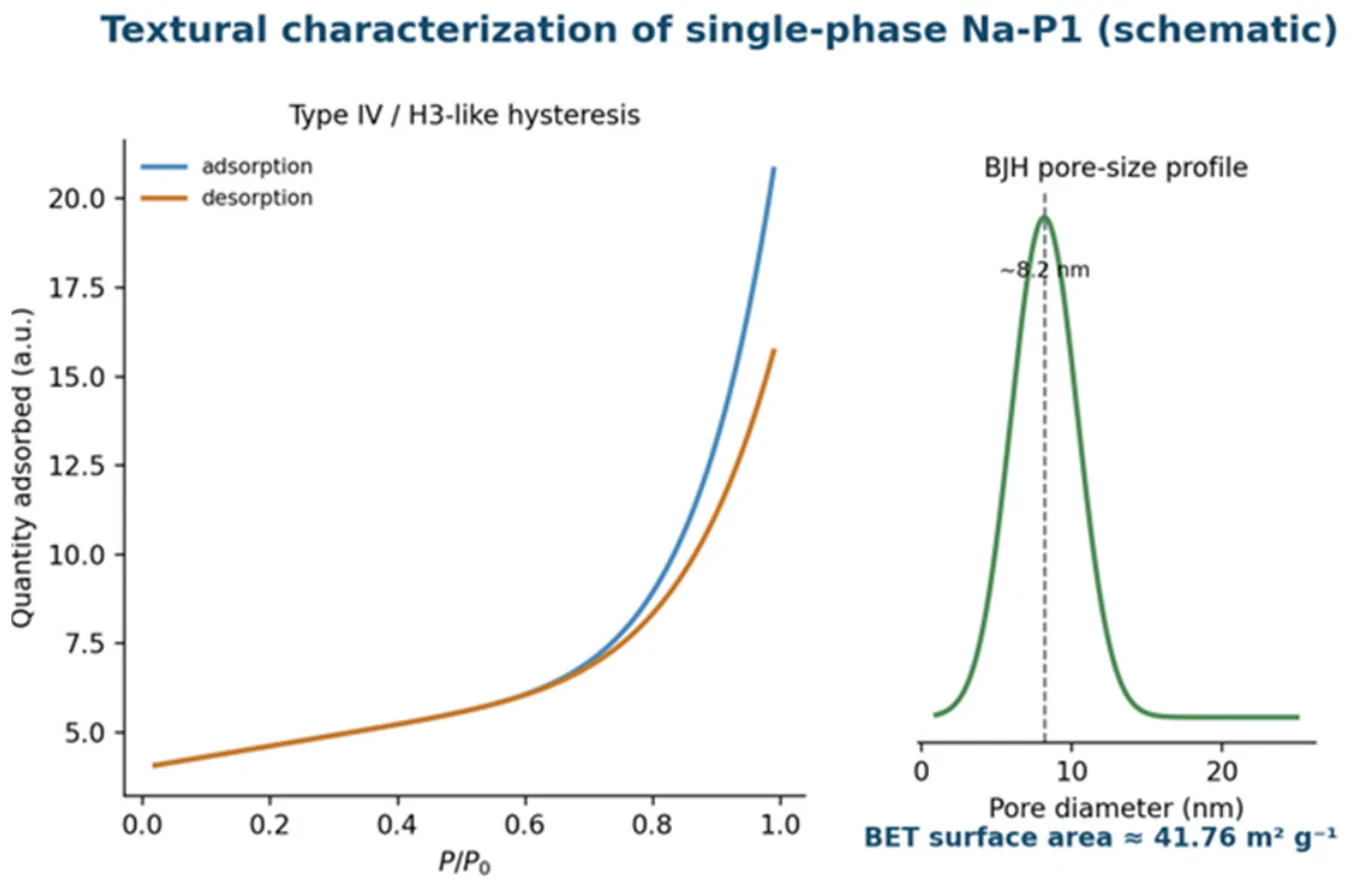
Original schematic of the reported textural behavior of single-phase Na-P1 synthesized from the Volclay fraction >2 µm at Si/Al = 1.34. The diagram summarizes the type IV/H3-like N_2_ adsorption–desorption behavior, BET surface area of approximately 41.76 m^2^ g^−1^, and BJH average pore diameter of approximately 8.2 nm reported by Boudia et al. [14]; it is not a reproduction of the published data plot.

For the clay-derived single-phase Na-P1 reported by Boudia et al. [14], a type-IV/H3-like N_2_ isotherm, BET area of ~41.76 m^2^ g^−1^, and BJH average pore diameter near 8.2 nm are best interpreted as evidence of secondary textural mesoporosity. The intrinsic GIS micropores remain present by crystallographic definition even when they are poorly sampled by N_2_ at 77 K. (Figure 7). (Table 6).

Taken together, XRD, SEM, bulk/solution chemistry, sorption, MAS NMR, and surface spectroscopy provide complementary evidence. Apparent disagreements should not automatically be assigned to “pore blockage”: low gas-accessible area can result from cation gating, probe-size limitations, residual amorphous matter, aggregation, or true pore obstruction. Multi-technique interpretation is therefore required before linking structure to adsorption performance.

#### 3.2.7. Solid State ^29^Si and ^27^Al Magic Angle Spinning Nuclear Magnetic Resonance (NMR)

Solid-state ^29^Si and ^27^Al magic-angle-spinning nuclear magnetic resonance (MAS NMR) probes local framework environments that are not resolved by diffraction alone. In GIS materials, ^29^Si resonances report the number of neighboring Al atoms and can support framework Si/Al estimates, whereas ^27^Al distinguishes predominantly tetrahedral framework Al from higher-coordinate or extra-framework contributions. Hydration state must be controlled because it can strongly affect line widths and apparent coordination [39,40].

**Figure 8:**
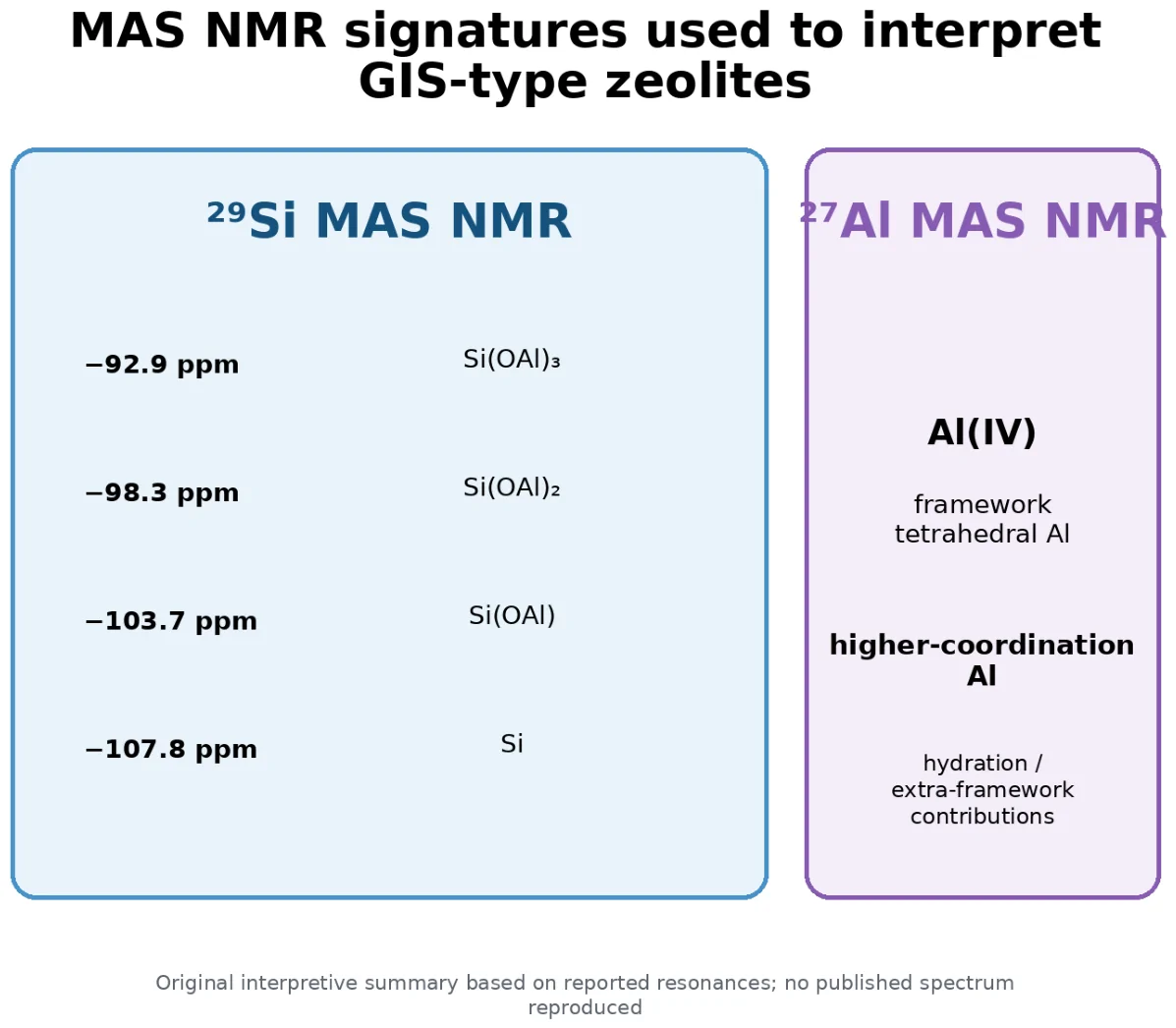
Original interpretive summary of ^29^Si and ^27^Al MAS NMR signatures used for GIS-type zeolite characterization. The ^29^Si chemical shifts and assignments follow the values discussed in the text for fly-ash-derived GIS zeolite [41]; the ^27^Al panel summarizes the distinction between tetrahedral framework Al and higher-coordination/extra-framework contributions. No published spectrum is reproduced.

Fly-ash-derived GIS spectra reported in the literature show multiple ^29^Si environments consistent with Q^4^(nAl) sites; the interpretive values summarized in Figure 8 are retained as local-structure markers rather than as unique fingerprints of phase purity [41].

The principal value of MAS NMR in this review is therefore to test whether bulk Si/Al changes correspond to framework substitution and to detect extra-framework Al generated by synthesis, ion exchange, or thermal treatment.

#### 3.2.8. X-Ray Photoelectron Spectroscopy (XPS) Analysis

X-ray photoelectron spectroscopy (XPS) provides surface-sensitive elemental and chemical-state information. For GIS materials it is most useful for following cation exchange, surface enrichment/depletion of Si and Al, metal loading, and changes in oxygen or hydroxyl environments after adsorption or modification. Because its sampling depth is surface-limited, XPS results should be distinguished from bulk composition measured by XRF/ICP-OES [42,43].

Direct GIS-specific XPS studies remain limited; the technique is consequently treated as a complementary surface probe rather than as a primary criterion for P1/P2 identification.

#### 3.2.9. Research Scope in GIS-Type Zeolites Characterization: EPMA and AFM

##### Electron Probe Micro-Analyzer (EPMA)

Electron probe microanalysis (EPMA) can map micrometer-scale Si, Al, Na, and impurity distributions and is particularly useful for testing chemical heterogeneity in waste-derived crystals. Its role is complementary to XRF because spatially resolved composition can reveal unreacted precursor domains or phase segregation that a bulk average can conceal [44].

GIS-specific EPMA datasets remain sparse, making spatially resolved precursor-to-product compositional mapping a useful future direction.

##### Atomic Force Microscopy (AFM)

Atomic force microscopy (AFM) could add nanoscale surface-topography and roughness information, but its use for GIS-type Zeolite-P remains rare. Rather than repeating general AFM principles, we note it here as an underused tool for resolving crystal-growth steps, external-surface defects, and morphology changes after adsorption/regeneration.

## 4. Preparation of Zeolite GIS

So far, a range of physicochemical and solvothermal methods have been employed and refined to synthesize GIS-type Zeolite-P, including hydrothermal, alkali-fusion, sol–gel, and alkali-leaching techniques. The choice of synthesis method is largely dependent on the specific zeolite form to be produced. The following subsections outline the various synthesis routes for Zeolite-P using industrial and agricultural waste-derived raw materials.

### 4.1. Hydrothermal Synthesis

Hydrothermal crystallization is the most widely used route to Zeolite-P and is the reference against which assisted methods are compared. For waste-derived feeds, the decisive variables are precursor dissolution, NaOH concentration, Si/Al and water content, aging, temperature, and time; autoclave operation and subsequent washing also contribute materially to energy and water demand. The method is therefore operationally mature but not automatically low-impact.

As reported by Adjdir and coworkers [14], another technique used to influence the synthesis outcome was the introduction of GIS-type Zeolite-P2 crystal seeds into a conventional Zeolite-P synthesis. These Na-P2 seeds were stored in a glass (GL) reactor (stage III GL alteration) and, when introduced, facilitated the crystallization of the GIS Zeolite-P1 polymorph by bypassing the nucleation stage, allowing crystal growth to commence immediately.

Higher temperature and longer treatment can accelerate conversion but also favor denser competing phases; phase-selective synthesis therefore requires balancing nucleation and growth rather than simply maximizing severity [7,45].

**Figure 9:**
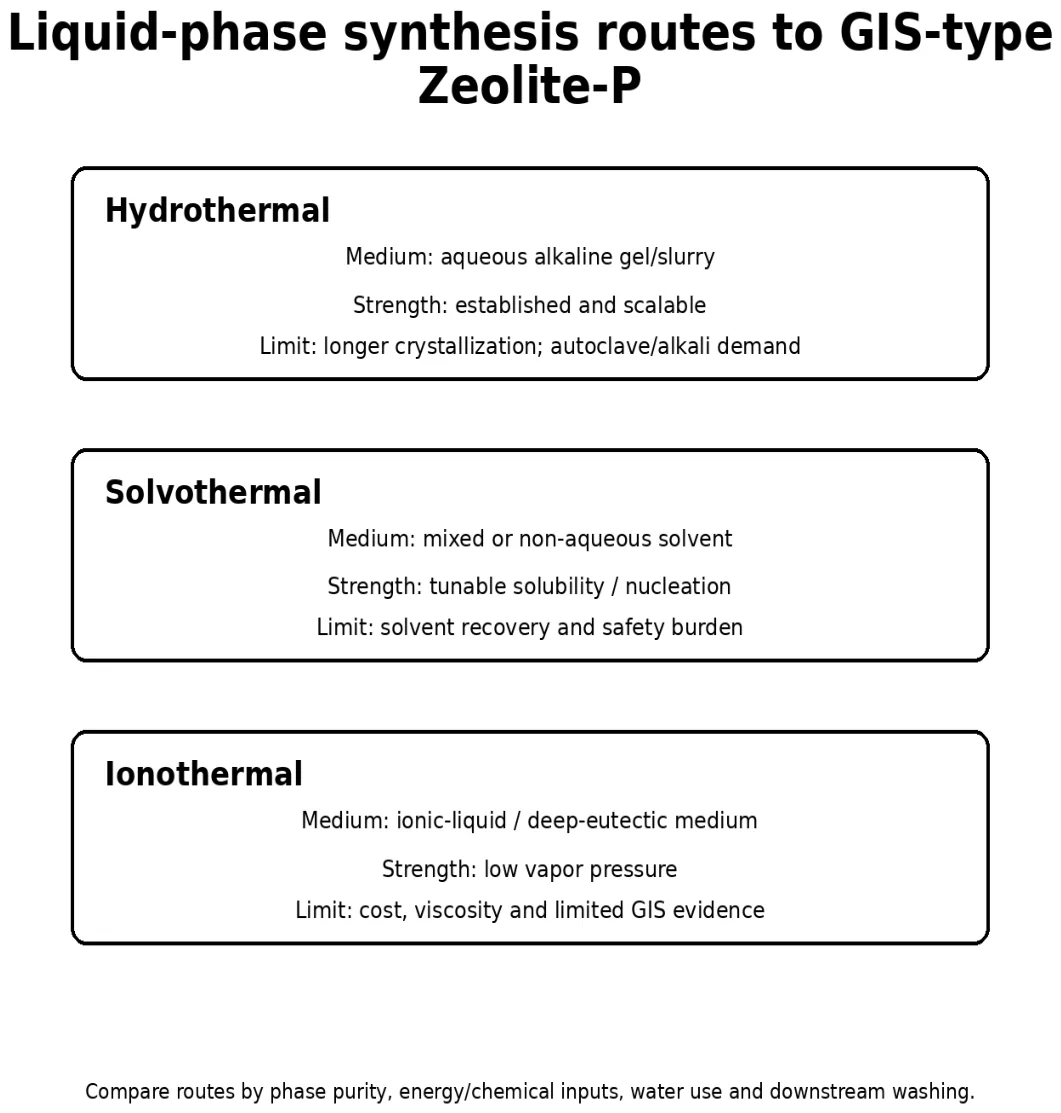
Standardized comparison of hydrothermal, solvothermal, and ionothermal liquid-phase routes. The figure emphasizes solvent environment and process trade-offs rather than presenting any route as intrinsically greener; phase purity, chemical/energy inputs, water use, and downstream washing should be assessed together [16].

Seed addition and precursor activation can shorten induction times and improve phase control, but the benefits should be evaluated against added preprocessing and washing steps. This trade-off motivates the comparisons with alkali fusion and energy-assisted routes below. (Figure 9).

### 4.2. Alkali-Fusion Hydrothermal Method

**Figure 10:**
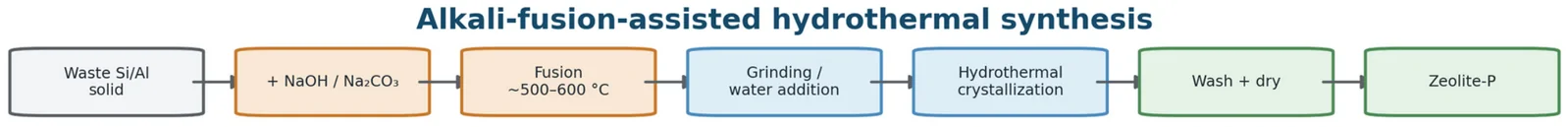
Original process schematic of Zeolite-P synthesis by the alkali-fusion-assisted hydrothermal route: waste-derived Si/Al precursor, alkali addition, fusion, water addition/grinding, hydrothermal crystallization, washing, and drying.

The alkali-fusion method is a well-established technique for Zeolite-P synthesis, particularly effective in extracting silica and aluminum from solid wastes, typically present in inert minerals such as the clay fraction > 2 µm. In this process, sodium hydroxide (NaOH) is commonly used as an activator to convert these components into soluble aluminate and silicate salts. Unlike solvothermal methods, in which alkali is used in solution as a mineralizer, the alkali-fusion process involves fusing the raw material with alkali in the solid state prior to any solvent treatment, followed by hydrothermal conditions for crystallization. (Figure 10). (Table 7).

Numerous studies have synthesized GIS-type Zeolite-P using this method. For instance, Adjdir and coworkers reported the synthesis of polymorphs P1 and P2 via alkali fusion followed by hydrothermal treatment using inert minerals from the clay fraction > 2 µm. This approach was successfully applied to low-grade raw materials, producing high-purity and anhydrous Zeolite-P [14]. Several comparative studies have consistently demonstrated the superiority of the alkali fusion-assisted hydrothermal route over conventional hydrothermal synthesis. Díaz and coworkers [13] showed that alkali fusion reduced metakaolinization time from 3 to 1 h, improved crystallinity from 75% to 84%, and increased cation-exchange capacity (CEC) from 250 to 300 mg(CaCO_3_) g^−1^. In line with this, Ge and coworkers [67] reported significant differences in the yield of Zeolite-P depending on the synthesis route: 50.34% by the fusion method (Na_2_CO_3_:fly ash ratio 1:1.2; 550 °C, 2 h fusion), 59.28% by the hydrothermal method (2 M NaOH; 7 h at 80 °C), and a higher yield of 70.52% by the alkali fusion-assisted hydrothermal method. Their findings also highlighted the contrasting effects of the alkali activators NaOH and Na_2_CO_3_ on Zeolite-P crystallization. Earlier, Yoshida and coworkers [68] highlighted the limitations of conventional hydrothermal synthesis, in which Zeolite-P formation was often considered unsuccessful due to the co-formation of hydroxysodalite, quartz (Qtz), and mullite (Mul), with only a minor fraction of the desired Zeolite-P phase obtained. By contrast, the alkali fusion step was shown to amorphize Qtz and Mul in fly ash, increase the Si/Al ratio, and thereby promote the crystallization of Zeolite-P. Furthermore, hydrothermal synthesis without fusion failed to convert feldspar (Kfs) effectively, thereby restricting zeolitization and limiting improvements in CEC (69 cmol(+) kg^−1^) due to its low reactivity. Consequently, the fusion–hydrothermal method not only ensured higher yields but also produced Zeolite-P with enhanced CEC (410 cmol(+) kg^−1^), attributed to the complete dissolution of reactive components, particularly Qtz and Kfs. (Table 8).

### 4.3. Sol–Gel Method

**Figure 11:**
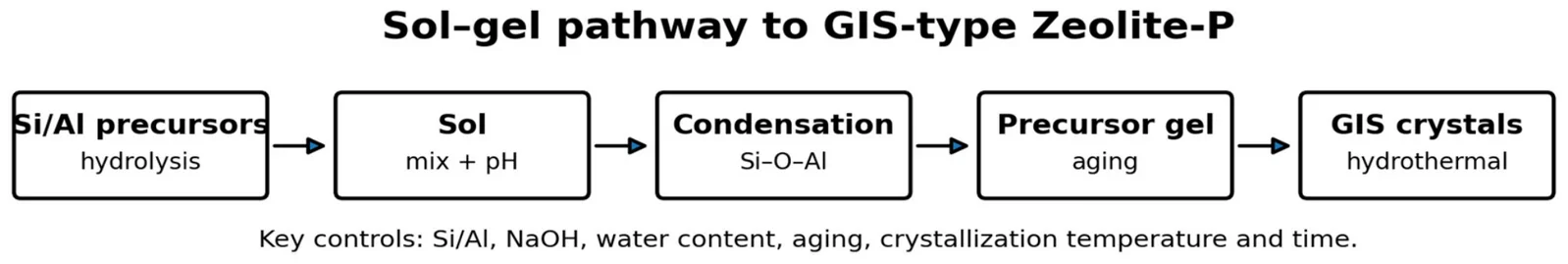
Standardized sol–gel sequence for GIS-type Zeolite-P: precursor hydrolysis/dissolution, sol formation, Si–O–Al condensation, precursor-gel aging, and hydrothermal crystallization. “Precursor gel” denotes the aged aluminosilicate network prior to crystallization.

In the sol–gel route, molecular or colloidal Si/Al precursors are hydrolyzed and condensed into an aluminosilicate gel before crystallization. The principal advantage is control over precursor homogeneity and gel chemistry; the principal limitation for low-cost waste valorization is that highly reactive or organometallic precursors can offset the economic benefit of the final material. Si/Al, alkalinity, water content, aging, and crystallization conditions remain the key phase-control variables. (Figure 11).

For instance, Wongkasemjit and coworkers reported the direct synthesis of zeolite GIS from alumatrane and silatrane precursors via a sol–gel process. Optimal gel transformation occurred at 110 °C for 3 h with a Na_2_O:SiO_2_ molar ratio of 3:1 and a SiO_2_/Al_2_O_3_ ratio of 4 [72].

Sol–gel synthesis can produce compositionally uniform materials, but claims of low energy or low cost are route-specific and should include precursor manufacture and solvent/alkoxide handling. For waste-derived Zeolite-P, direct hydrothermal or activated-waste routes generally have a stronger scale-up evidence base. (Table 9).

While conventional solution-based methods have laid the foundation for GIS-zeolite synthesis, recent advances in unconventional energy-assisted techniques have enabled faster crystallization and higher-purity Zeolite-P, thereby enhancing material quality.

### 4.4. Microwave Method

**Figure 12:**
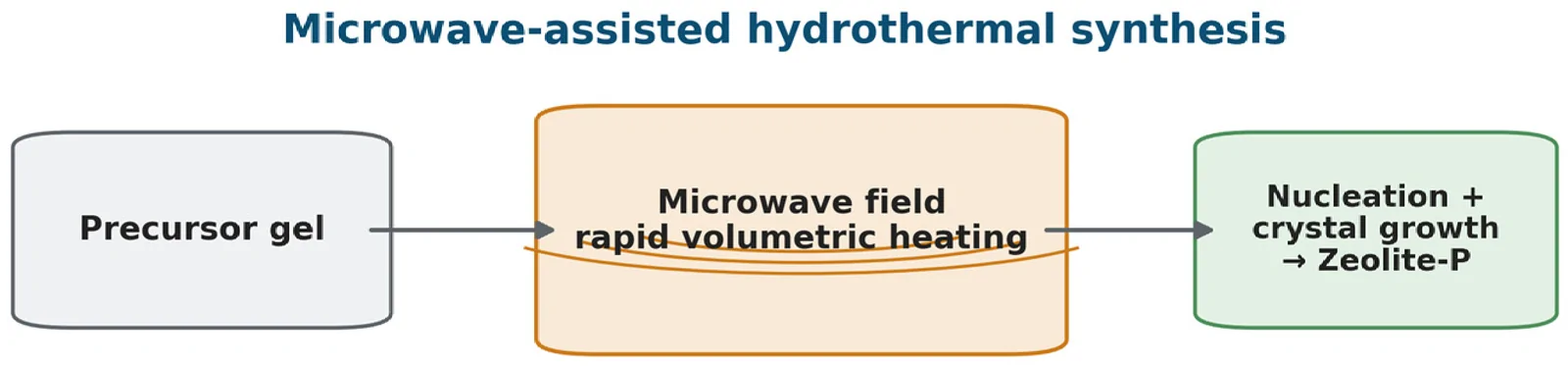
Original process schematic of microwave-assisted hydrothermal synthesis of Zeolite-P, emphasizing rapid volumetric heating followed by nucleation and crystal growth.

Microwave-assisted hydrothermal synthesis uses volumetric dielectric heating to shorten heat-up and crystallization times. Reported benefits include faster nucleation and smaller crystals, but scale-up depends on field uniformity, penetration depth, reactor design, and actual electrical energy per unit product. Consequently, shorter synthesis time should not be equated automatically with lower life-cycle impact. (Figure 12).

A comparative study was conducted by Wittayakun and co-workers between conventional hydrothermal (CH) and microwave-assisted hydrothermal (MH) synthesis methods for extracting silica from cogon grass and using it in zeolite GIS production. Since the CH method was relatively slow, the MH approach was explored to reduce the synthesis time. The results showed that MH synthesis produced Zeolite-P with higher purity, attributed to rapid and uniform heating, whereas CH synthesis yielded Zeolite-P with greater crystallinity, larger particle size, higher BET surface area, and increased total acidity [78]. (Table 10).

In the context of waste-derived GIS-type zeolite, Long and co-workers synthesized Zeolite-P1 from municipal solid waste incineration fly ash using a microwave-assisted hydrothermal method. This approach proved to be energy-efficient, environmentally friendly, and cost-effective, requiring no pretreatment steps such as water washing, acid washing, or alkali fusion. The synthesis was conducted at 180 °C for varying durations (0.5–2 h). Under optimal conditions, the resulting Na-P1 zeolite exhibited a high BET surface area of 61.42 m^2^/g and a substantial total pore volume of 0.44 cm^3^/g [85].

### 4.5. Ultrasound Energy Method

Ultrasound-assisted synthesis uses acoustic cavitation to intensify mixing, dissolution, and nucleation. It can shorten crystallization for some waste-derived systems, but scale-up, probe/bath energy efficiency, equipment wear, and reproducibility remain important constraints. The technique is therefore best evaluated as process intensification rather than intrinsically “green” synthesis. (Table 11).

Solvent-free and trace-water routes can reduce liquid handling and wastewater generation, but their overall environmental advantage still depends on precursor activation, alkali loading, temperature, yield, and post-treatment.

### 4.6. Solvent-Free Synthesis of GIS Zeolite

Solvent-free or trace-water crystallization reduces bulk solvent use and can increase reactor productivity. For waste-derived GIS, this is attractive where hydrated salts or residual moisture supply sufficient water for ion transport; however, “solvent-free” does not eliminate the impacts of alkali production, heating, grinding, or product washing.

Several studies demonstrated the feasibility of synthesizing GIS-zeolite under solvent-free conditions. Xiao and co-workers synthesized silicoaluminophosphate zeolite with a GIS framework through a solvent-free strategy in which raw materials were mechanically mixed and then heated in a closed vessel without the need for additional solvents [88]. Wu and coworkers reported the preparation of zeolite P1 from fluidized-bed fly ash using a trace-water solvent-free process [89]. In that study, a solid mixture of fly ash and Na_2_SiO_3_·9H_2_O with a mole ratio of Na:Al:Si = 1.5:1:1.7 was transformed into zeolite P1 at 80 °C within 24–96 h, without adding sodium hydroxide. The product exhibited a higher adsorption capacity (22.9 mg g^−1^) than many natural zeolites (Zeo) and comparable performance to hydrothermally synthesized Zeo, while also showing excellent thermal stability up to 943 °C. In a related work, high-purity zeolite P was synthesized from fly ash via a solvent-free route at 120 °C over 72 h, in which only trace water was required, leading to zero wastewater discharge [90]. More recently, Kong and co-workers produced GIS-type zeolite via OSDA/solvent-free methods by varying the Si/Al ratio and crystallization temperature. At higher Si/Al ratios and elevated temperatures, Zeolite-P was obtained, underlining the process flexibility and controllability offered by solvent-free methods [91].

Reported solvent-free GIS syntheses demonstrate technical feasibility and, in some cases, reduced wastewater generation. These advantages should be confirmed by mass/energy balances and life-cycle assessment before the route is ranked as environmentally superior to hydrothermal synthesis. (Table 12).

From a scale-up perspective, conventional hydrothermal and alkali-fusion-assisted hydrothermal routes currently have the broadest waste-precursor evidence base. Microwave and ultrasound can reduce reaction time, while solvent-free and sol–gel approaches offer specific process advantages, but comparisons should be made using phase yield/purity, energy, alkali and water consumption, waste treatment, and shaped-adsorbent performance rather than crystallization time alone.

### 4.7. Novel Methods and Trends in GIS Zeolite Synthesis

Advances in GIS-type zeolite synthesis are pivotal in expanding their applications across emerging and conventional processes. Beyond conventional, microwave-, and ultrasound-assisted methods, recent advances have further accelerated the discovery of GIS-type zeolitic structures. These methods include interzeolite and topotactic conversions, as well as ionothermal synthesis [92]. Interzeolite conversions involved the transformation of zeolite A into Zeolite-P [93], natural clinoptilolite (Cpt) zeolite into synthetic GIS zeolite by exploiting shared structural units [64], which crystallized as polymorphs P1 or P2 [94], and faujasite framework (FAU)-to-GIS transformation [95]. In particular, Adjdir and co-workers reported a reversible intrazeolitic transformation between the P1 and P2 zeolite phases [14]. Furthermore, Thoma and Neno synthesized zeolite GIS via a vapor-phase transport (VPT) crystallization method, starting from sol–gel-derived inorganic precursor gels. The gels were exposed to vapor (not in direct contact with liquid) and were heated at 175 °C for 7–14 days. The vapor interacted with the gel, leading to the crystallization of Zeolite-P [96]. In addition, alternative activation methods were developed to address the limitations of conventional alkali fusion. Nagasaka and coworkers synthesized Zeolite-P from fly ash using a wet-grinding-assisted hydrothermal method [97]. This mechanochemical–hydrothermal synthesis process reduced energy consumption by nearly 46% and improved relative crystallinity to 89.37%. Zeolite P2 was also crystallized from zeolite P1 via a seed-assisted hydrothermal method in a glass (GL) reactor, producing additional silica and increasing the Si/Al ratio, consistent with stage III GL alteration [98]. To date, no reports exist on GIS-zeolite synthesis via topotactic transformation through selective removal of labile atoms followed by pillarization, or through ionothermal synthesis using ionic liquids instead of water. Nevertheless, ionothermal synthesis has shown promise in related zeotypes. For example, nano-sized NaY zeolite composites were successfully synthesized from metakaolin [99], and silicoaluminophosphate zeotypes with LTA structure (SAPO-LTA) were prepared using the ionic liquid 1-ethyl-3-methylimidazolium ([emim]^+^) [100]. These alternative methods offer new opportunities for developing and tailoring GIS-type Zeolite-P with enhanced structural and functional properties.

## 5. Diagram of Kinetic Phases in GIS Polymorph Crystallization

**Figure 13:**
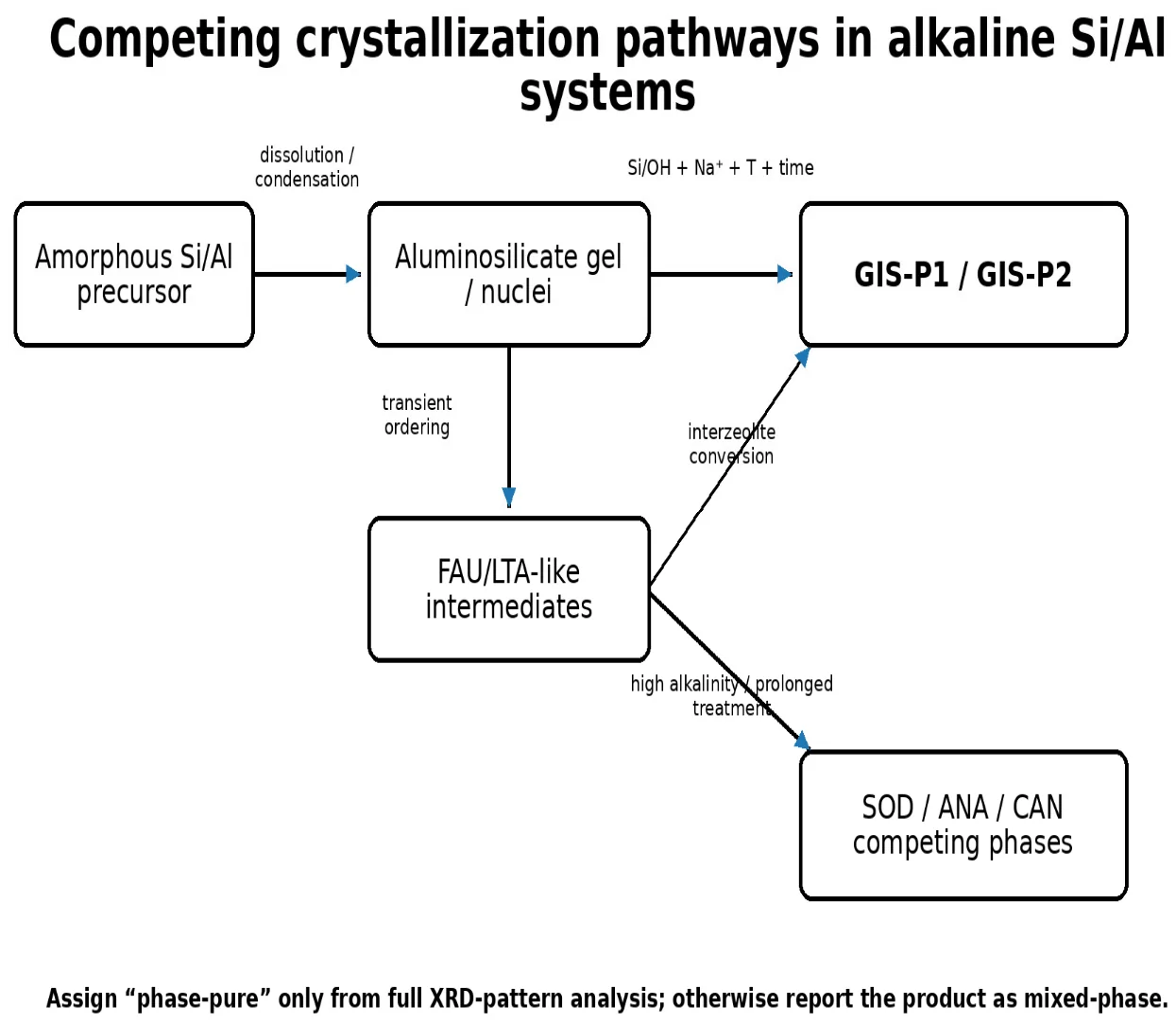
Standardized schematic of competing crystallization pathways in alkaline Si/Al systems. The diagram highlights transient aluminosilicate/FAU-LTA-like intermediates, GIS-P1/P2 formation, and competing denser phases (e.g., SOD/ANA/CAN) without implying a universal single pathway. Phase purity must be established by full XRD-pattern analysis [7,101].

**Figure 14:**
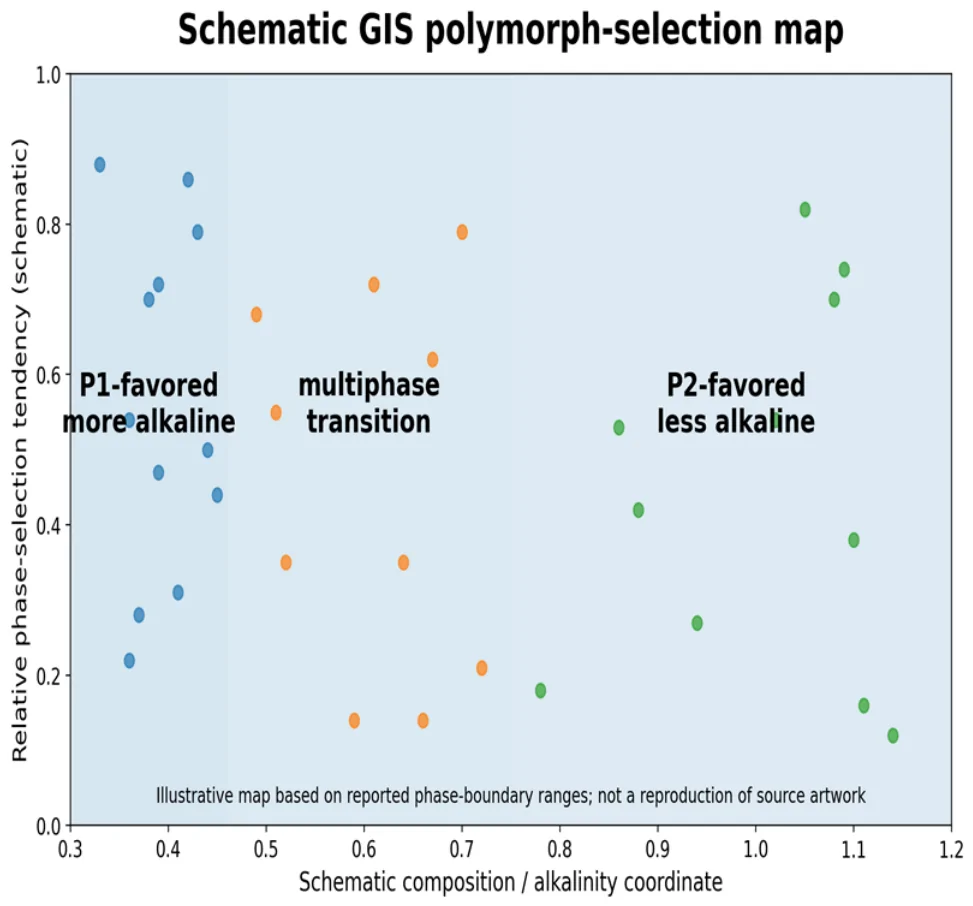
Original schematic GIS polymorph-selection map based on the reported phase-boundary ranges of Oleksiak et al. [7]: Na-P1 is favored at lower Si/OH values (<0.46), Na-P2 at higher Si/OH values (>0.75), with a multiphase transition region between them. The plotted points are illustrative only and do not reproduce the source phase diagram.

The formation of GIS polymorphs P1 and P2 requires precise control over key parameters, such as alkalinity, as measured by the Si/OH molar ratio and the Si/Al ratio, to achieve pure phase formation. Rimer and coworkers [7] systematically investigated the growth conditions for these polymorphs by varying the Si/Al ratios (1.5–9.0) and Si/OH ratios (0.3–1.2) and synthesizing samples at 100 °C for 7 days. By constructing a kinetic phase diagram based on powder XRD data, they identified distinct regions corresponding to the single phase of Na-P1 or Na-P2, as well as multiphase zones containing impurities such as sodalite (SOD), faujasite (FAU), and/or analcime (ANA), Figure 13 and Figure 14. The phase boundaries between P1 and P2 were demarcated by regions of differing Si/OH ratios, with Na-P1 forming in more alkaline conditions (Si/OH < 0.46) and Na-P2 forming in less alkaline conditions (Si/OH > 0.75). While P1 and P2 exhibit similar bulk crystal morphologies, their unique powder XRD patterns enable unambiguous structural identification [7]. Notably, P2 has been rarely reported in the literature, with most studies either neglecting to include XRD data or misidentifying the crystal structure as a generic GIS-type zeolite. (Figure 13). (Figure 14).

Previous studies explored the structural transformation between P1 and P2; however, the findings of Rimer and coworkers suggested that this transformation was not universal [7]. Time-resolved studies revealed that FAU often preceded the crystallization of GIS, highlighting the FAU-to-GIS interzeolite transformation as a critical kinetic pathway. The formation of pure GIS phases was favored in Si-rich solutions, whereas high Al content promoted the formation of SOD or LTA. Although the phase diagram provided valuable insights, the presence of multiphase regions complicated the clear delineation of boundaries between pure GIS polymorphs. Additionally, temperature, heating time, and water content significantly impacted the kinetic phase diagram, with higher or lower temperatures favoring the formation of other zeolite (Zeo) structures, such as FAU, ANA, or cancrinite [102]. This diagram contributed to a deeper understanding of GIS polymorph crystallization and offered a framework for tailoring synthesis conditions to obtain a specific polymorph of choice.

The Si/OH and Si/Al conditions delineated above provide a predictive basis for anticipating which polymorph, Na-P1 or Na-P2, a given waste-derived synthesis route is likely to yield. Where reported by the original authors, polymorph identity is indicated alongside the corresponding performance data in the tables of Section 7, Section 8, Section 9 and Section 10, since P1 and P2 differ measurably in framework density and consequently in ion-exchange and sorption behavior; readers should treat this distinction as a relevant variable, not an incidental detail, when comparing results across studies.

## 6. Synthesis Cost of Zeolite-P from Waste-Derived Precursors

Economic and environmental comparisons of waste-derived Zeolite-P are complicated by inconsistent system boundaries. Feedstock purchase price is only one component: collection, transport, drying, milling, classification, washing, acid/alkali pretreatment, NaOH or other reagents, heating, reactor occupancy, solid–liquid separation, product washing/drying, effluent treatment, shaping, and regeneration can all be significant. Therefore, this review no longer assumes that waste precursors have zero or near-zero cost.

Cross-study cost values are also difficult to normalize because electricity price, reagent price, plant scale, yield, purity, year/currency, and whether capital depreciation or wastewater treatment is included vary widely. Table 13 is retained only as an illustrative input/energy checklist and should not be interpreted as a normalized techno-economic ranking between synthesis routes.

For adsorption applications, cost per kilogram of powder is an incomplete metric. More decision-relevant denominators include cost per mole of ion-exchange capacity, cost per kilogram of pollutant removed over the usable lifetime, and cost per treated volume at a defined breakthrough criterion. Shaping further changes delivered performance: industrial zeolitic adsorbents commonly contain ~5–30 wt.% binder, and inert binders can reduce accessible porosity and slow diffusion, although the magnitude is binder- and formulation-specific rather than a universal 10–30% penalty [16]. Recent life-cycle analysis of fly-ash-derived zeolite production confirms that environmental conclusions are strongly route- and functional-unit-dependent [103].

## 7. Industrial and Agro Wastes as Raw Materials for Zeolite-P Synthesis

### 7.1. Natural, Conventional Synthetic and Waste-Derived Zeolites: A Property-Based Comparison

Three material classes are relevant to the applications reviewed here: (i) natural zeolite ores, dominated by clinoptilolite, mordenite, chabazite and phillipsite [104,105]; (ii) conventional synthetic zeolites prepared from reagent-grade Si and Al sources; and (iii) waste-derived zeolites, in which the Si/Al source is an industrial, agro-industrial or mineral residue [106,107,108]. Gismondine itself is a rare mineral that is not exploited commercially, so the natural comparators differ in framework topology from Zeolite-P; Table 14 therefore compares material classes and is complemented, where data exist, by same-topology comparisons of Na-P prepared from waste and reagent precursors (Table 18 and Table 20).

Two points follow. First, the properties that separate the classes are set largely by framework type and Si/Al ratio rather than by the origin of the raw material: a reagent-derived and a waste-derived Na-P of identical Si/Al, phase purity and cation form are expected to behave alike in ion exchange, whereas clinoptilolite (Si/Al ≈ 4–5) differs fundamentally in exchange capacity, acid stability and acid-site chemistry [104,109]. Second, origin acts indirectly: it determines the available Si/Al ratio, the residual crystalline and amorphous phases, the trace elements and therefore reproducibility, leaching risk and the population of acid/base sites [15,107,108].

**Table 14:** Property-based comparison of natural, conventional synthetic and waste-derived zeolites relevant to ion exchange, adsorption and catalysis. Natural = clinoptilolite/mordenite-type ores; synthetic and waste-derived = GIS-type Na-P. Values are typical literature ranges or calculated ideal values and depend on deposit, precursor and route.

Property	Natural Zeolite Ores	Conventional Synthetic Na-P	Waste-Derived Na-P
Framework/phases	HEU, MOR, CHA, PHI-type phases, usually mixed; gismondine rare [18,104,105]	Single, designed phase: Na-P1 (cubic) or Na-P2 (tetragonal), selected by Si/OH ratio and alkalinity [7,14]	Na-P1/P2 as target, but co-crystallizing SOD, ANA, CAN and FAU/LTA-type phases are common; route and seeding decide the outcome [7,101]
Crystallinity/phase purity	Zeolite content deposit-dependent (typically ~50–90 wt.%); quartz, feldspar, clays, volcanic glass	Typically high (>90–95%) and reproducible	Wide range; unreacted quartz/mullite/glass and amorphous gel remain if dissolution is incomplete [14,107,108]
Si/Al ratio	≈4–5 (clinoptilolite, mordenite)	≈1–2; tunable through gel composition	≈1.1–1.9 reported; fixed by precursor Si/Al plus added Si or Al (1.34 Na-P1; 1.81 Na-P2 from clay fraction [14])
Surface area and porosity	Micropores (8–12-ring); N_2_ BET often only tens of m^2^ g^−1^ because cations and impurities restrict access	GIS 8-ring channels (≈3 × 4.5 Å); N_2_ (77 K) access poor; BET area mostly external/interparticle	Same intrinsic microporosity; mesoporosity is secondary (aggregation, residues), e.g., 41.76 m^2^ g^−1^ and 8.2 nm for Na-P1 [14]; Table 6
Purity/residual phases/trace elements	Site-dependent quartz, feldspar, clay, Fe oxides; possible trace metals	Minimal; limited to reagent impurities	Mullite, quartz, hematite/magnetite, Ca/Mg/Fe/Ti-bearing phases; possibly hazardous trace metals—leaching tests mandatory [15]
Ion-exchange capacity (CEC)	≈2.2 meq g^−1^ theoretical (clinoptilolite, ideal formula); practical values lower	Up to ≈4.6–5.6 meq g^−1^ theoretical for hydrated Na-P (Si/Al 1.7–1.0); practical value set by accessibility	Same ceiling (framework Al) multiplied by zeolite fraction; diluted by non-zeolitic mass and blocked by residues
Hydrothermal/chemical stability	High; tolerates acid and several hundred °C better than Al-rich phases [104]	Stable in neutral–alkaline media; Al-rich framework dealuminates/amorphizes in acid and on harsh thermal treatment	As conventional; residual alkali, Ca/Fe phases and low crystallinity may alter stability; test in real water chemistry
Acid/base sites	H-form accessible (higher Si/Al); Lewis sites from cations and Fe/Ca impurities; usually used in Na/K/Ca form	Na-form: negligible Brønsted, weak cation-Lewis, basic framework O; H-form hard to obtain (acid-labile) [110]	As conventional plus impurity-derived Lewis/redox sites and more silanol/Al–OH defects from amorphous residue; not yet systematically quantified (Section Acid–Base Sites in GIS-Type Zeolite-P and the Influence of Precursor Origin and Synthesis Route)
Reproducibility/scale	Deposit-dependent; no synthesis step	High, but reagent-intensive	Precursor lot variability; extra pretreatment (milling, fusion); scale-up evidence limited (Table 12)
Cost/environmental profile	Lowest cost; mining and beneficiation impacts	Reagent, NaOH, energy and water inputs	Low-cost feed, but alkali, energy, water and washing remain; LCA data scarce (Section 6)
Best-suited process	Bulk NH_4_^+^, Cs/Sr removal, low-cost polishing, agriculture	Ca/Mg softening (detergent builder), phase-pure reference, gas-adsorption studies	Ca/Mg/heavy-metal removal and CO_2_ studies where residue valorization is a goal and leaching is controlled

Practical implications. Waste-derived Na-P is advantageous when (a) high exchange capacity for Ca^2+^, Mg^2+^, NH_4_^+^ or divalent heavy metals in neutral–alkaline media is required, (b) an Al-rich, controllable framework is wanted without reagent-grade precursors, and (c) valorization of a local residue is a design objective. It is disadvantageous when (a) drinking-water or food-contact use is intended without certified phase-purity and leaching control [15], (b) acidic or high-temperature conditions are expected, because Al-rich GIS is acid-labile, or (c) strong Brønsted acidity is needed, which favours high-silica frameworks such as MFI or BEA [109,111].

Natural zeolites remain preferable for bulk, low-cost treatment where moderate capacity and compositional variability are acceptable [104,105]; conventional synthetic Na-P is preferable where reproducibility and phase purity dominate, for example in reference studies of gas adsorption or in detergent builders; waste-derived Na-P is justified when the first two criteria can be met at acceptable cost and the residue valorization benefit is real. A fair test of the waste origin requires same-topology comparisons at matched Si/Al, phase purity and cation form, which remain scarce (Section Acid–Base Sites in GIS-Type Zeolite-P and the Influence of Precursor Origin and Synthesis Route).

**Figure 15:**
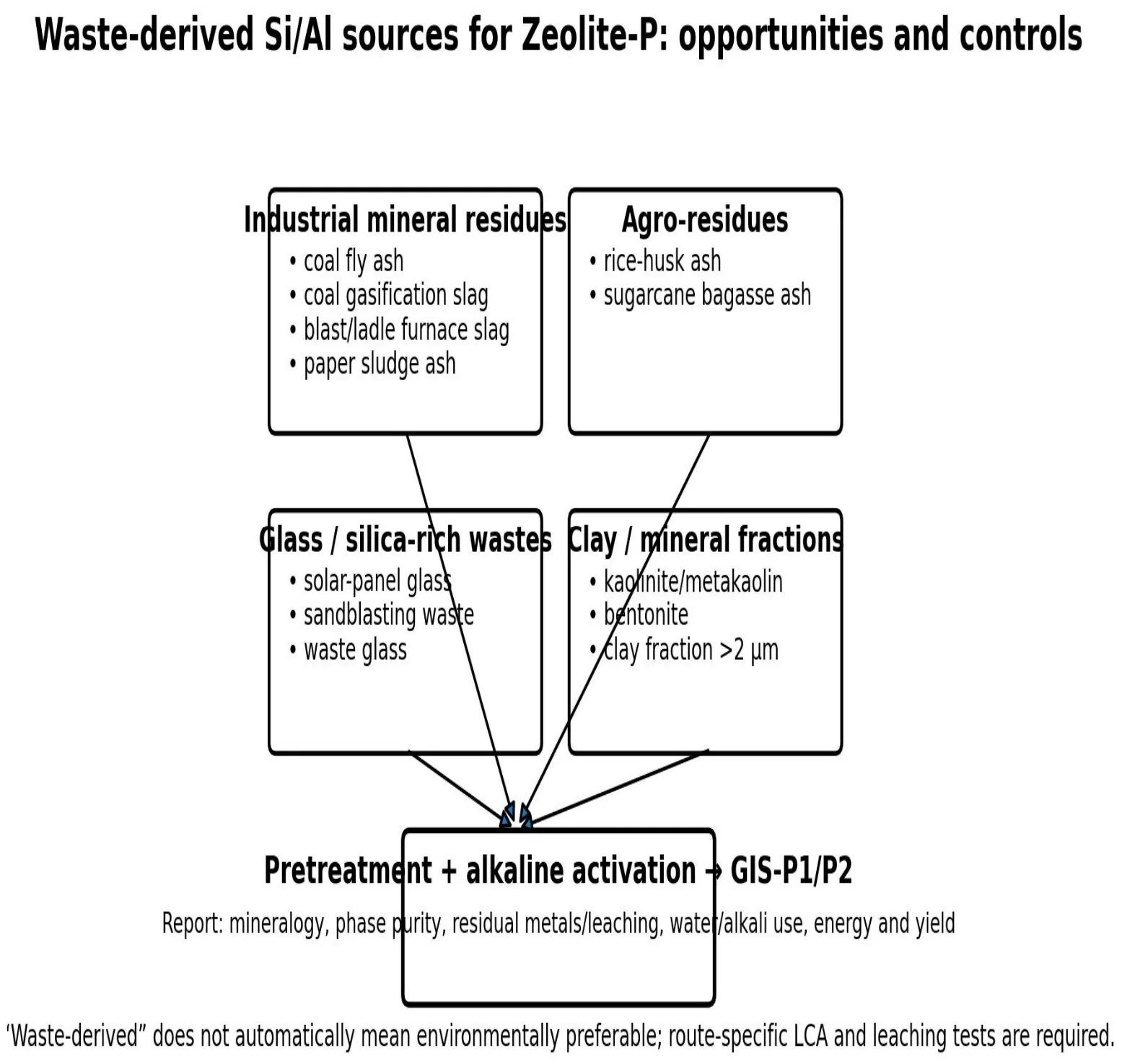
Representative industrial, agro-industrial, glass, and clay/mineral Si/Al sources used for Zeolite-P synthesis. The revised figure also highlights the controls required for meaningful sustainability claims: precursor mineralogy, phase purity, residual-metal/leaching tests, alkali/water consumption, energy use, and product yield.

Waste valorization can reduce virgin Si/Al demand and landfill burden, but residues such as fly ash, gasification slag, and end-of-life glass can contain trace metals or soluble salts. Conversion to a zeolite does not automatically eliminate these hazards. Studies intended for water treatment should therefore report feed and product composition, standardized leaching under relevant pH/ionic-strength conditions, and the fate of contaminants during synthesis, washing, use, and regeneration. This requirement is especially important when the adsorbent itself is produced from a hazardous waste stream [15]. (Figure 15). (Table 15).

### 7.2. Coal Fly Ash

As coal continues to dominate electricity generation, the resulting increase in coal-based industrial waste, particularly coal fly ash (CFA), has become a significant environmental concern [106]. CFA, a fine particulate byproduct formed during coal combustion in thermal power plants, accounts for about 70–90% of the total ash generated during coal combustion. It is typically collected from flue gases through electrostatic or mechanical precipitation techniques [121,122]. With its amorphous (AM) silica–aluminate structure, CFA presents an industrial opportunity for conversion into value-added products, addressing both environmental and health challenges. Its toxic trace element content renders it a pollutant, and improper storage in waste collection sites poses serious health risks to nearby ecosystems and human populations [123]. Typically gray or black in color, CFA possesses alkaline properties, particle sizes ranging from 1 to 100 μm, and a large specific surface area of 0.2–10 m^2^/g. Its chemical composition includes elements such as Si, Al, Ca, and Fe, with oxides of these elements forming key compounds. Crystalline minerals in CFA, including aluminosilicates (Als) and mullite (Mul) (Al_2_O_3_·2SiO_2_), as well as quartz (Qtz) and iron oxides (IO) (magnetite”Mag” Fe_3_O_4_ and hematite”Hem” Fe_2_O_3_), make CFA a potentially valuable resource for reuse [124,125,126]. During coal combustion, approximately 0.15 ton of CFA is produced per ton of coal [127], highlighting the scale of waste generated and the need for innovative solutions to mitigate its environmental impact. This has spurred research into its potential as a raw material for synthesizing value-added products [128,129], particularly Zeolite-P. The first successful synthesis of Na–P zeolite from Class F fly ash was reported by Grutzeck and co-workers in 1992 [130], where crystallization occurred after 2 days of aging at room temperature followed by 6 days of curing at 80 °C in NaOH solution. Notably, no P-type zeolites formed when KOH was used, underscoring the crucial role of sodium ions in the process. Since then, numerous studies have refined this synthesis of Na-P1 zeolite from coal fly ash by optimizing hydrothermal conditions, such as alkali concentration, liquid-to-solid ratio, temperature, and duration, using methods like the Taguchi design [131], to maximize yield and crystallinity. Recent research has focused on advanced activation and energy-assisted techniques for synthesizing GIS-zeolite from fly ash (FA). For instance, Farhadi and coworkers reported that the crystalline structure of fly ash could be collapsed via alkaline fusion at 550 °C for 1 h using an FA/NaOH weight ratio of 1/1.2. This produced a synthesis solution with a molar composition of 2.2 SiO_2_:Al_2_O_3_:5.28 Na_2_O:106 H_2_O, which was subsequently hydrothermally treated to crystallize Zeolite Na–P1 [69]. To address the high energy demand of alkaline fusion, Nagasaka and coworkers proposed wet-grinding activation as a lower-energy alternative. This method effectively destroyed the insoluble phases in FA and enabled the synthesis of Na–P with a relative crystallinity of 89.37% without any aging step [97].

Furthermore, Rohani and coworkers transformed CFA to Zeolite-P using a sonochemically assisted route, which shortened the crystallization time from 24 h to 4 h while maintaining high crystallinity (87% at a NaOH/CFA ratio of 0.27) [31,132]. Subsequently, they demonstrated that coal fly ash could also be converted to Zeolite-P under atmospheric pressure by combining 6 h of conventional heating with 30 min of microwave irradiation, achieving a crystallinity of 97% at a CFA/NaOH ratio of 0.40 g/mL. In addition, to overcome the limitations associated with the fusion of impurities in raw FA within trace water systems, Wu and co-workers reported that fluidized bed FA with a molar ratio of Na:Al:Si = 1.5:1:1.7 could be successfully converted to Zeolite-P1 at 80 °C within 24–96 h via a solvent-free process [88]. For further details on CFA-derived Zeolite-P synthesis, given its importance, readers are referred to the “Raw Materials” column in the Tables in Section 5 and Section 8.

### 7.3. Coal Gasification Slag

Coal gasification, a high-temperature process used to convert coal into syngas, produced two distinct byproducts: fine slag collected from filters and coarse slag (CS) obtained from lock chambers [133]. As coal underwent rapid decomposition during gasification, the mineral components were transformed into molten slag. This molten slag adhered to the gasifier walls and flowed to the furnace bottom, where it solidified into coarse slag [133]. Simultaneously, smaller particles were transported by the airflow, purified along with the synthesis gas, and subsequently formed fine slag. The primary components of coal gasification slag included SiO_2_, Al_2_O_3_, CaO, Fe_2_O_3_, and residual carbon [134]. Concentrations of key inorganic elements, including Si, Al, K, Na, Ca, Mg, Fe, and S, ranged from 5 to 132 mg/g, while trace elements such as Cu, Zn, Ni, Co, Zr, Pb, Ti, Cr, Y, Rb, As, Mn, Ba, and Sr were found in amounts between 37 and 4063 μg/g. Particle sizes typically varied between 1.13 and 5.16 mm, indicating potential for recovery and utilization in industrial applications [135,136]. These valuable components and elements, present in both coarse and fine slag, underscored the importance of coal gasification slag as a resource for Zeolite-P synthesis. For instance, Ma and coworkers., in 2020 [137], Ji and coworkers., in 2021 [138], Xing and coworkers., in 2023 [139], Bai and coworkers., in 2023 [140]; Zhou and coworkers., in 2023 [141]; Ma and coworkers., in 2024 [113]; Li and coworkers., in 2024 [142]; Guo and coworkers., in 2024 [143] successfully derived Zeolite-P from coal gasification slag.

### 7.4. Agro-Wastes

#### 7.4.1. Rice Husk Ash

Rice husks, the hard protective outer layer of rice grains, contain a significant amount of silica (15–28% by weight) and are separated during the milling process, particularly in rice-producing regions [144]. Rice husk ash (RHA), produced by burning these husks, typically exhibits a grayish-black color due to residual unburned carbon. When combusted at temperatures between 550 and 800 °C, RHA primarily contains amorphous (AM) silica, while higher temperatures result in crystalline silica formation. The silica content in RHA ranges from 85 to 98% by weight, with purity highly dependent on factors such as combustion conditions, rice variety, and regional climatic and geographical influences on rice cultivation [145]. With a specific gravity between 2.11 and 2.27, RHA is characterized by its lightweight, highly porous structure and elevated specific surface area [146]. Rice husk is generally unsuitable for animal feed because of its low nutritional value and poor digestibility. Common disposal practices such as long-term storage and open-field incineration contribute to significant environmental issues, including energy wastage, greenhouse gas emissions, air pollution, and land use concerns [144]. However, the high silica content of rice husk has spurred research into its potential to produce Zeolite-P. For instance, Wittayakun and coworkers, in 2008 [147], Adil and coworkers, in 2010 [148], Naskar and coworkers, in 2013 [52], Naskar and coworkers, in 2014 [149], Thi-Tran-Anh and coworkers, in 2023 [150], and di Profio and coworkers, in 2024 [151], reported the successful synthesis of Zeolite-P from rice husk-derived materials. This repurposing of rice husk into a sustainable material addresses both waste management and resource utilization challenges.

#### 7.4.2. Sugarcane Bagasse Ash

Sugarcane is the most widely produced crop globally, generating substantial quantities of wet bagasse as a byproduct during the sugar extraction process. The disposal and management of this biomass waste pose significant environmental challenges. The most common method of handling it is incineration, which results in another byproduct, sugarcane bagasse ash (SCBA) [152]. SCBA, an industrial waste of agricultural origin, is primarily composed of silicon dioxide (SiO_2_), with varying chemical compositions depending on its source. Typically, it contains 53–91% silica and 12–31% alumina oxides [153]. Beyond construction applications, the SiO_2_ and Al_2_O_3_ content of SCBA has made it a promising, cost-effective raw material for the production of Zeolite-P. For instance, Shah and coworkers in 2011 [154], Shah and coworkers in 2012 [155], and Hayat and coworkers in 2019 [115] reported the successful synthesis of Zeolite-P from SCBA. This approach not only offers an environmentally friendly solution for managing SCBA waste but also contributes to the development of valuable materials in a sustainable manner.

### 7.5. Sandblasting and Solar Panel Glass Wastes

As global demand for energy rises, the depletion of fossil fuel reserves such as coal, natural gas, and oil, coupled with their environmental impacts, has accelerated the search for alternative energy sources. Solar energy stands out as a promising technology due to its renewable and environmentally friendly nature [156]. However, the rapid growth in solar energy capacity brings challenges related to the disposal of solar panel waste at the end of their lifespan, typically 25–30 years. The accumulation of solar panel waste in landfills poses environmental concerns, particularly due to the presence of toxic materials like lead, and it also results in the loss of valuable materials, necessitating recycling efforts [116]. More than 80% of silicon-based solar panels are composed of glass (GL), and as the industry progresses, technologies aimed at reducing silicon usage, such as thin-film solar cells and advanced cutting techniques, are being developed. Nevertheless, these processes generate significant amounts of sandblasting waste (SW), which contains high concentrations of silicon, alumina, and silicon carbide, all of which require proper disposal. The composition of both GL waste and SW, rich in silica and alumina, has sparked interest in their use as raw material sources for zeolite (Zeo) synthesis. Given that zeolites are composed of silicates and aluminates, this approach aligns with sustainability efforts, and recent studies have explored the potential for converting these wastes into valuable zeolitic materials [117]. For instance, Lin and coworkers, in 2022 [157] and Lin and coworkers, in 2023 [118], reported the synthesis of Zeolite-P derived from solar panel glass waste and sandblasting waste.

### 7.6. Clay Fraction Greater than 2 µm for Zeolite-P Synthesis

The increasing demand for silicon- and aluminum-based materials has led to significant accumulation of clay fractions greater than 2 µm (Cf > 2 µm) through sedimentation processes. This often underutilized clay fraction, rich in aluminosilicate (Als) minerals, presents a promising opportunity as a raw material for Zeolite (Zeo) synthesis, particularly in an environmentally friendly context. The composition of Cf > 2 µm typically includes minerals such as quartz (Qz: 3.9–4.9 wt.%), plagioclase (Pl: 3.3–4.9 wt.%), orthoclase (Or: 3.1–4.9 wt.%), gypsum (Gp: 3–4.2 wt.%), muscovite (Ms)/illite(Ilt)(1.9–3.7 wt.%), and calcite (Cal: 0.9–1.7 wt.%). A unique example of such syntheses was reported for the first time in 2023 by Adjdir and coworkers [14], resulting in the successful crystallization of high-purity Zeolite-P using Cf > 2 µm of volclay. Their research highlighted the potential for large-scale industrial applications of low-cost zeolite production from abundant clay fractions greater than 2 µm. This approach not only enhanced the utilization of an often neglected resource but also contributed valuable insights into hydrothermal synthesis processes, furthering the development of sustainable materials.

### 7.7. Some Other Sources for Si and Al in Zeolite-P Synthesis

In addition to coal fly ash, coal gasification slag, agro-wastes, and glass (GL) waste, other slags can also serve as sources of Si and Al for zeolite (Zeo) production. Zeolite-P was prepared from paper sludge ash [47,119], blast furnace slag [120], and ladle furnace slag [158]. Its water vapor adsorption capacity was comparable to that of commercial Zeolite-P.

On the other hand, in 1998, El Maaoui and coworkers prepared Na-P1 zeolite from interstratified illite–smectite (Ilt–Sme) [159]. Metakaolin is also rich in SiO_2_ and Al_2_O_3_. After their alkaline fusion, the kaolin (Kn) was used to produce Zeolite-P via the hydrothermal method [160]. Moreover, aluminum solid waste was recycled as an Al source for Zeolite-P synthesis. For instance, López-Andrés and coworkers designed a one-step synthesis of Zeolite-P from hazardous aluminum solid waste [56]. In addition to inorganic materials, certain organic sources have also been explored. Prasetyoko and coworkers reported a hydrothermal study of mesoporous Zeolite-P synthesis using Sapindus rarak extract as a natural surfactant [161]. Furthermore, some inorganic precursor solutions were used to prepare knobby-surfaced, mesoporous, and single-phase Zeolite-P [162].

Across these precursor classes, alkaline reactivity broadly tracks the amorphous or readily dissolvable Si/Al fraction. However, precursor mineralogy alone does not predict final performance: phase purity, cation form, particle aggregation, residual phases, and secondary porosity can dominate adsorption. Waste-derived products should therefore be compared on a phase- and application-specific basis rather than by feedstock category alone.

## 8. Potential Differences in the Structural Characteristics and Performance of Waste-Derived Zeolite-P

Table 16 illustrates the wide spread in textural and adsorption properties reported for Zeolite-P/GIS materials obtained from different feeds and routes. These values should not be interpreted as a simple ranking of precursor quality because the studies differ in polymorph/phase purity, probe molecule, cation form, synthesis severity, and adsorption protocol. Where the cited work did not quantify phase purity, the entry is treated as “GIS/Na-P as reported” rather than assumed to be pure P1 or P2. Mesopore diameters are reported in nm and pore volumes in cm^3^ g^−1^ for consistency.

The strongest transferable conclusion is therefore mechanistic: precursor dissolution controls the chemistry available for nucleation, whereas synthesis route controls phase competition, crystal size, aggregation, and accessible texture. Performance comparisons require these variables—and not only BET area or a single adsorption capacity—to be reported together.

### Acid–Base Sites in GIS-Type Zeolite-P and the Influence of Precursor Origin and Synthesis Route

Acid–base chemistry controls catalytic activity and also influences adsorption through proton exchange, pH buffering, hydrolysis of polyvalent cations and CO_2_–framework interactions [109,111]. In Na-P (Si/Al ≈ 1–2) the framework charge is fully compensated by Na^+^, so Brønsted acidity is negligible and the framework oxygens are strongly basic; Lewis character arises from the exchangeable cations, from extra-framework Al and from non-zeolitic impurities [110]. Because next-nearest-neighbour Al atoms weaken the acid strength of a site, Al-rich GIS cannot match the acid strength of high-silica frameworks such as MFI or BEA, even when protons are introduced [109,111]. Furthermore, Al-rich frameworks are acid-labile, so NH_4_^+^-exchange/calcination can partly amorphize or dealuminate the material, converting Brønsted precursors into extra-framework Al.

The amount, strength and distribution of these sites depend on origin and preparation in several ways: (i) Si/Al ratio, set by the precursor and by added Si or Al, fixes the maximum number of framework charges and the Al–O–Si environment [167]; (ii) cation type and hydration matter because Ca^2+^, Mg^2+^ and Fe^3+^, abundant in fly ash, slag and clay residues, can generate Brønsted-type protons by hydrolysis of their hydration shell, whereas Na^+^ and K^+^ act as weak Lewis centres; (iii) crystallinity and residues matter because unreacted glass or amorphous gel adds weak silanol/Al–OH sites, while Fe/Ti-bearing phases add Lewis/redox sites unrelated to the GIS framework; (iv) synthesis route can alter the amount of extra-framework species, residual alkali, crystal size and external silanols; and (v) washing, ion exchange and calcination change the cation inventory and basicity.

Acidity is typically quantified by NH_3_-TPD and by FTIR of adsorbed pyridine, with Brønsted and Lewis sites distinguished by the ≈1545 and ≈1450 cm^−1^ bands [168,169,170]; a recent study on phosphorus-modified Zn/La/Fe-ZSM-5 illustrates how combining these two methods shows that moderating strong Brønsted acidity and adding Lewis sites changes selectivity and coking in alkane aromatization [171]. For GIS, however, pyridine is excluded from the 8-ring channels and the method reports only external sites; smaller probes, ^1^H and ^27^Al MAS NMR, and CO_2_-TPD for basic sites are needed, and activation and hydration state must be matched across samples. On the available evidence, the performance of waste-derived Zeolite-P in the adsorption applications reviewed here is governed by framework Al content, cation form, phase purity and accessible texture rather than by waste origin per se; origin acts through those variables and through impurity-related sites. A systematic, matched comparison of site densities in waste- and reagent-derived GIS is still missing (Section 11). (Table 17).

## 9. Structuring and Shaping of GIS-Type Zeolitic Adsorbents for Industrial Applications

Industrial adsorbents must be shaped from fine powders into pellets, beads, extrudates, monoliths, or other bodies that provide acceptable pressure drop, attrition resistance, heat/mass transfer, and handling. Shaping can therefore change the adsorption behavior measured for the parent powder and must be considered part of adsorbent design rather than a downstream packaging step [16,172,173].

Binder choice is especially important for small-pore GIS because nonporous clays, silica, or alumina can dilute the active phase, partially block interparticle transport paths, or alter diffusion. Reviews of industrial zeolite shaping report final binder contents commonly in the ~5–30 wt.% range, with the capacity/kinetic penalty strongly dependent on binder chemistry, particle size, porosity, and calcination history rather than on a single universal percentage [16]. Binderless or binder-converted bodies can mitigate these losses but add processing steps.

Shaping methods for zeolitic gismondine (GIS) adsorbents include:

(a) *Granulation*: This method forms small spherical beads or pellets (usually < 5 mm in diameter) from the powder by adding water through droplets or sprays. The resulting beads are often uniform and are suitable for various applications [16].
(b) *Extrusion*: In this process, Zeolite-P powder is mixed with water to form a paste, which is then forced through a die under pressure to produce polyhedral shapes, often cylindrical. This method is commonly used to create monoliths—larger, single-piece structures that contain macroscopic holes for faster mass transfer and reduced pressure drop during use [16].
(c) *3D printing*: This emerging technology allows the formation of zeolitic GIS adsorbents with highly customizable shapes [174]. While still predominantly at the laboratory scale, 3D printing holds promise for producing advanced materials with specific properties tailored to industrial needs [175].

After shaping, drying/calcination establishes mechanical integrity but adds energy demand and can modify cation location, hydration, and accessible porosity. For waste-derived Zeolite-P, future studies should report powder and shaped-body performance side by side, including binder fraction, crush/attrition strength, pressure drop, adsorption kinetics/capacity, regeneration, and cost per delivered treatment capacity.

## 10. Properties and Applications of Synthetic Zeolite GIS

**Figure 16:**
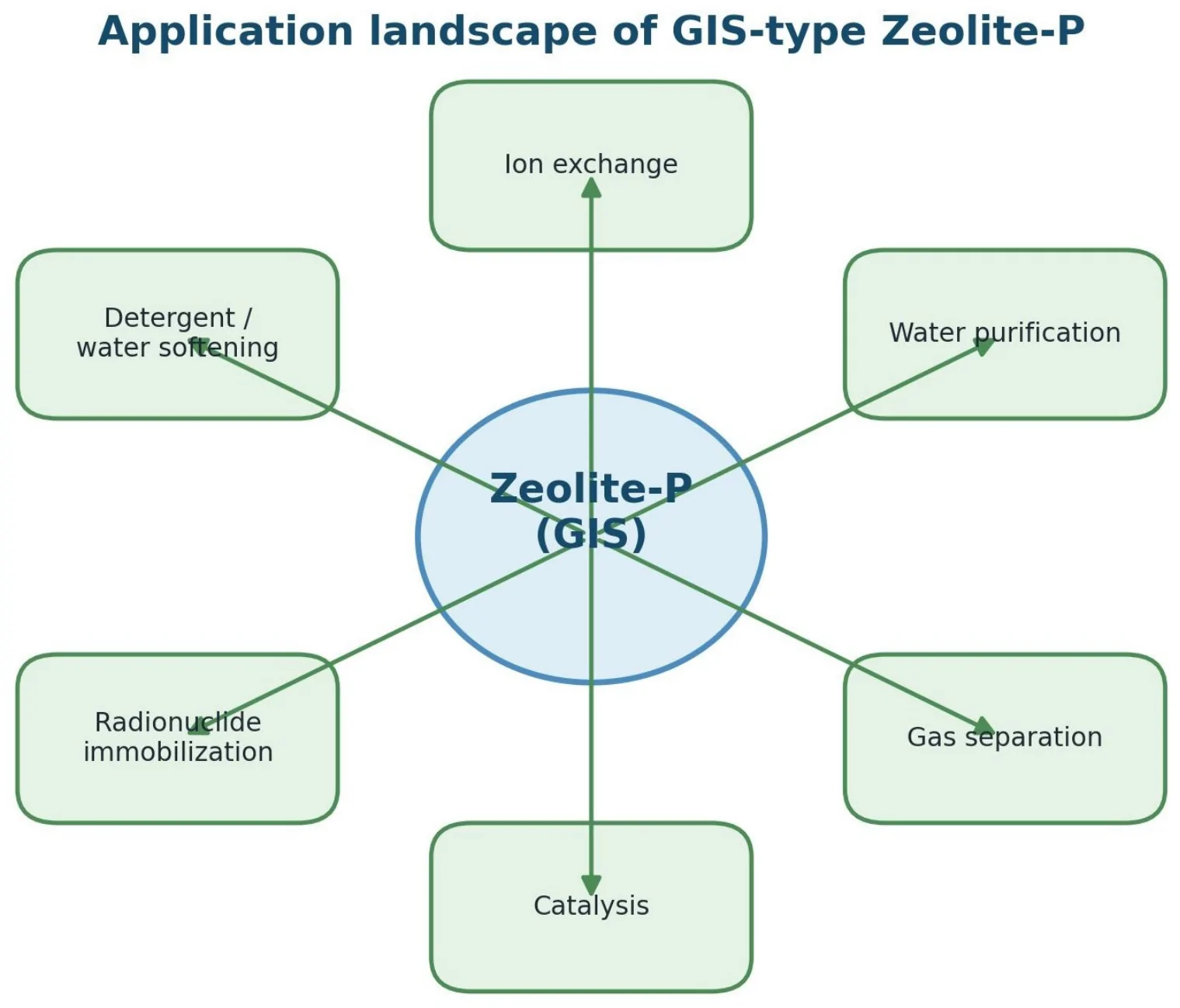
Original schematic of major application areas of GIS-type Zeolite-P, including ion exchange, water purification, gas separation, catalysis, radionuclide immobilization, and detergent/water-softening applications, based on the literature reviewed in Section 10.

GIS polymorphs differ markedly in stability and accessibility. P2 is generally more thermally robust than P1, whereas high-Al Na-P forms provide high charge density and ion-exchange capacity but can suffer stronger cation gating and restricted diffusion [7]. These trade-offs explain why GIS is well established for ion exchange and water adsorption but remains less broadly deployed in high-temperature catalysis or gas separation. (Figure 16).

### 10.1. Ion Exchangers

Zeolites (Zeo) have proven to be potential ion exchangers for industrial applications, while a synthetic Zeo with a gismondine (GIS) structure, commercialized in 1995, demonstrated improved performance for water softening in laundry detergents [176]. Additionally, in 2002, ion exchange was investigated in Zeolite MAP, a synthetic GIS used as a commercial detergent builder [177].

Ion-exchange studies of Zeolite MAP/GIS have examined monovalent and divalent cations including Li^+^, Na^+^, K^+^, Rb^+^, Cs^+^, Mg^2+^, Ca^2+^, Sr^2+^, and Ba^2+^. Reported selectivity trends depend on hydration and steric accessibility; the flexible GIS framework can reorganize during exchange, and large cations can be kinetically restricted [177,178].

Furthermore, a notable challenge in ion-exchange measurements arises when using low-silica Zeo in dilute solutions, as the immediate release of sodium ions can significantly alter the pH, complicating the assessment of exchange properties [179]. Beyond ion exchange, zeolite GIS also facilitates filtration, surface precipitation, and adsorption, enabling the removal of anions, organic contaminants, and particulate matter from water sources, thus broadening their utility in water treatment applications [180].

### 10.2. Catalytic Applications

Catalysis is a secondary, less mature application of GIS-type Zeolite-P. Structure–activity relationships depend on the Si/Al ratio, the balance of Brønsted/Lewis or basic sites, exchanged cations, crystal size, and accessibility of reactants to the small GIS windows. Low-Si/Al Na forms are naturally rich in exchangeable/basic cations, whereas conversion to a stable protonic acidic form can be constrained by dealumination or loss of crystallinity during thermal treatment. Metal-loaded or composite GIS materials can add redox or interfacial functionality, but in such systems the active phase must be distinguished from the zeolite support.

Reported examples include Zeolite-P-containing composites for transesterification/biodiesel production [181] and metal-containing GIS materials. These studies indicate that activity can be tuned by cation identity and composite chemistry, but the evidence base is far smaller than for MFI, FAU, or BEA. Because pore accessibility and thermal stability can dominate turnover and deactivation, catalytic comparisons should report phase purity, Si/Al ratio, acid/base-site characterization, exchanged cation/metal loading, crystal size, and reuse under matched reaction conditions. The article title has therefore been revised so that catalysis is no longer presented as a co-equal application axis.

### 10.3. Gas-Phase Adsorption and Selective Separation

Early computational screening of idealized, often pure-silica GIS frameworks predicted high CO_2_ uptake/selectivity [182,183,184]. These calculations identified energetically favorable sites in the GIS cages and windows, but they did not include the full Al/Na chemistry of realistic Zeolite-P. GIS should therefore be described here as an adsorbent or separation medium, not as a catalyst, for CO_2_/N_2_ or CO_2_/CH_4_ separation.

Experimental work on realistic Na-P1/Na-P2 provides an important correction to the idealized predictions. Oleksiak et al. [7] combined adsorption measurements with density-functional calculations and found that both GIS polymorphs are strongly selective for H_2_O relative to light gases, while measured CO_2_ uptake at near-ambient conditions was far below the values predicted for pure-silica GIS. The discrepancy is consistent with restricted access through small windows, extra-framework Na^+^ occupancy, framework Al, hydration, and cation–sorbate interactions. Low apparent Ar/N_2_ BET areas in some Na-GIS samples likewise need not imply absence of crystallographic microporosity.

Water, with its strong interaction with framework charge and cations, shows much higher uptake than weakly interacting light gases. Secondary meso-/macroporosity in aggregated Na-P1 microspheres can further increase high-relative-pressure water uptake by capillary condensation, but that contribution is distinct from adsorption in the intrinsic GIS micropores [23].

More recent engineered GIS materials show that composition and crystal engineering can improve gas-separation performance under selected conditions. Deng et al. [185] reported CO_2_ uptake of 3.5 mmol g^−1^ and a volumetric uptake of 128 cm^3^(STP) cm^−3^ at 195 K, whereas Al Atrach et al. [186] reported dynamic CO_2_/N_2_ and CO_2_/CH_4_ separation for nanosized and micron-sized GIS samples. Such results should not be generalized across GIS materials without matching cation form, temperature, pressure, mixture composition, bed geometry, and activation protocol.

Overall, the gas-separation literature supports a more nuanced conclusion than earlier screening studies: GIS is highly relevant to dehydration and can be engineered for selected gas separations, but idealized pure-silica predictions are not directly transferable to aluminosilicate Na-P1/P2. Reported H_2_, CO_2_, N_2_, and CH_4_ uptake should therefore be interpreted together with cation population, framework composition, crystal size, and kinetic accessibility.

### 10.4. Aqueous-Phase Adsorption and Sorption Tests on Zeolite-P

For aqueous treatment, the key performance metrics are not adsorption capacity alone but selectivity in the target matrix, kinetics, regeneration, structural/leaching stability, and performance in a continuous bed. Batch data obtained from single-solute model solutions are valuable for mechanistic comparison but should not be presented as directly representative of industrial wastewater without testing competing ions, dissolved organic matter, ionic strength, and pH effects.

In the following sections, “adsorbent” is used for the solid phase and “adsorbate” for the retained species. GIS micropores can contribute ion exchange and size-selective transport, while external surfaces and secondary mesoporosity can contribute additional binding and faster transport. For large hydrated ions and dyes, uptake may occur substantially on external surfaces, interparticle porosity, or composite phases rather than by penetration of the intrinsic GIS channel system.

Reported batch studies vary widely in pH, concentration, solid/liquid ratio, contact time, and temperature, so direct rankings of qmax values can be misleading. Where possible, this review emphasizes matched operating conditions and distinguishes model-water studies from multicomponent or real-water tests.

Crystal size and hierarchical secondary porosity can reduce mass-transfer limitations, but they do not change the fundamental microporous classification of the GIS framework. Modifications that create mesopores, reduce crystal size, or alter cation form should therefore be reported separately from intrinsic framework pore size.

The distribution of framework Al and extra-framework cations controls charge density and ion exchange; in practice, however, precursor impurities and composite phases can add additional surface-complexation or precipitation pathways. Mechanistic assignments should thus be supported by post-adsorption spectroscopy/chemistry rather than inferred from capacity trends alone.

#### 10.4.1. Zeolite GIS-Based Adsorbents

Zeolite adsorption research established molecular sieving and cation exchange as central separation mechanisms [187]. For practical Zeolite-P selection, the relevant criteria are selectivity, usable capacity at realistic concentrations, kinetics, ease of regeneration, structural/mechanical durability, leaching safety, and cost per treated volume—not a single equilibrium capacity.

Gismondine (GIS) zeolites have emerged as highly effective adsorbents ideal for a range of separations, primarily due to their chemical and structural flexibility, microporosity, uniform pore size, and robust thermal stability. Thermodynamic separations operate at equilibrium, leveraging differences in interaction strength between adsorbates and the adsorbent. In contrast, kinetic separations occur away from equilibrium and depend on disparities in adsorption rates [188]. Molecular sieving, a unique mechanism exclusive to nanoporous materials like zeolite GIS, facilitates separation by excluding certain components based on their size and shape, making it an extreme form of kinetic separation. Herein, zeolite GIS in adsorption processes primarily focused on heavy metal, dye, and some organic contaminant removal, where zeolite GIS played a crucial role due to its mixed pores and capacity for size and shape selectivity, along with its tunable adsorption properties. The versatility and precision of zeolite GIS-based separations continue to drive their significance in both industrial and research contexts.

##### Heavy Metal, Cation and Anion Removal

GIS-type zeolites have been studied for heavy-metal and nutrient removal, principally through ion exchange and surface-associated processes. Reported targets include Ca^2+^, Cu^2+^, Cd^2+^, Cr^3+^/Cr^6+^, Zn^2+^, Ni^2+^, Mn^2+^, Pb^2+^, Cs^+^, Sr^2+^, Hg^2+^, Fe^3+^, NH_4_^+^, and phosphate. The very high capacities reported in some studies should be interpreted with the experimental concentration, pH, competing ions, and product composition shown in Table 17 and Table 18.

In 1993, Mimura and Akiba demonstrated that Zeolite-P had favorable adsorption properties for Cs and Sr. The saturated amounts of Cs and Sr were estimated to be 1.76 and 1.84 mmol/g, respectively [8]. In 2021, S. Hong et al. enhanced these properties when NaP zeolite, synthesized by a top-down desilication method, showed higher removal efficiency of Cs, Sr, and Ni: 35.3, 27.9, and 27.8 mg/g, respectively [63].

Zeolite-P exhibited a stable capacity of approximately 62.3 mg/g (298 K; C_0_, 230 mg/L) [60]. Ai and coworkers [29] studied the enhancement in Zn adsorption by as-synthesized zeolite NaP under the steric hindrance effect with the addition of 2-amino-methyl-propyl alcohol, polyethylene glycol (PEG), and cyclohexanol (CyOH) compared with raw NaP zeolite. Considering the studied adsorption conditions (pH 5, 120 min, 25 °C; adsorbent/adsorbate 50 mg/20 mL, 100 mg/L Zn^2+^), CyOH corresponded to the highest adsorption capacity: NaP-PEG 35.18 < NaP-RAW 36.55 < NaP-AMP 38.71 < NaP-CyOH 39.96 mg/g. Further, Zhang and coworkers [189] reported that through the Zn adsorption test of NaP zeolite before and after regulation with sodium dodecylbenzene sulfonate (SDBS) and cetyltrimethylammonium bromide (CTAB), the adsorption capacities of NaP zeolite for Zn followed this order: NaP-SDBS > NaP-RAW > NaP-CTAB.

Huang and coworkers demonstrated extraordinarily effective decontamination of heavy metals (Zn^2+^, Cu^2+^, Cd^2+^, and Pb^2+^) using renewable Zeolite-P. The maximum capacities of Zn^2+^, Cu^2+^, Cd^2+^, and Pb^2+^ were 88, 90, 210, and 649 mg/g, respectively (natural pH values, 5–1000 ppm, 25 °C, 250 min) [190].

Adil and coworkers [148] revealed that zeolite KP had a greater capacity to adsorb Ca^2+^ and Zn^2+^ ions than zeolite NaP and proved that potassium modification of zeolite NaP improved its adsorption capacities (68.03 → 120.48 mg/g for Ca^2+^; 106.38 → 138.89 mg/g for Zn^2+^). Fodial and coworkers [38] evaluated the sorption efficiency of synthesized NaP zeolite for Ca^2+^ from aqueous solutions under optimal conditions, comparing Na_2_O/SiO_2_ molar ratios of 1.13 and 1.10. They found that the NaP zeolite prepared with a Na_2_O/SiO_2_ ratio of 1.13 demonstrated superior calcium sorption capabilities. This enhanced performance was attributed to the higher proportion of acid sites present on the external surface of the Zeolite-P at this composition.

Additionally, the synthesized zeolite GIS presented a high adsorption capacity of 44.05 mg/g for the Mn^2+^ ion in aqueous medium at pH 8 with 30 min of contact time [84].

Zeolite NaP-based composite (3 wt.%), graphene oxide loaded into the interpores of hollow NaP zeolite, was investigated by Fodial and coworkers [35] for adsorption of ferric ions (Fe^3+^). It showed a maximum adsorption capacity of 103.0 mg/g from the aqueous solution.

On the other hand, Awwad and coworkers [12] applied the Zeolite-P-based composite (Bentonite/Zeolite-P) in the purification of sewage water and groundwater, based on their effective sequestration of ammonium ions (maximum experimental capacity of 155 mg/g at pH 6), achieving ammonium removal from groundwater and reducing ammonium ions in sewage water to 2.3 mg/L. Zeolite-P efficiently and continuously removed ammonium in solution through physical adsorption [164].

It is noteworthy that Bahiraei and Behin [59] reported a high adsorption capacity for mercury Hg removal, ca. 103.2 mg/g (pH 7, C_0_: 20 mg/L, 25 °C) via a NaP-zeolite-based loaded adsorbent (3.3 g/L of MnO_2_/NaP-zeolite).

Additionally, while Zeolite-P possesses a net negative charge that favors cation exchange, this property limits its ability to remove anionic species. Despite this, modified Zeolite-P, such as Zeolite-P-based composites and those treated with quaternary amines or aluminum, have demonstrated increased affinity for anions like phosphate and sulfate, expanding the utility of Zeolite-P beyond cation exchange to target inorganic oxyanions and organic pollutants. For instance, a Zeolite-P-based composite (Bentonite/Zeolite-P) in the purification of sewage water and groundwater, based on effective sequestration of phosphate ions (maximum experimental capacity of 170 mg/g at pH 6), achieved complete removal of phosphate from sewage water [12].

These results demonstrate strong laboratory potential, but most metal-ion datasets remain single-solute batch tests in model water. The smaller subset of groundwater, sewage, simulated-wastewater, multicomponent, and column studies is therefore more informative for translation and is discussed separately below. (Table 18). (Table 19).

**Table 18:** Heavy-metal adsorption capacities and experimental conditions reported for Zeolite-P/GIS materials. The table includes both waste-derived and reference/reagent-derived materials; matrix and phase-purity differences should be considered when comparing capacities.

Solid Waste Type	Adsorbates (Heavy Metals)	CEC (meq/100 g)	Initial Concentration (mg/L)	pH	S/L Ratios (g/mL)	Time (min)	Temperature (°C)	Adsorption Capacity q (mg/g)	Ref.
Natural mordenite (Mor) and clinoptilolite (Cpt)	Cs^+^ Sr^2+^	368	10	4.5	0.003	10 h	25	233.9 161.2	[8]
Natural Zeolite ores	Cs+ Sr^2+^ Ni^2+^	310.89	10	Neutral	0.01	12 h 24 h 24 h	Room temperature	35.3 27.9 27.8	[63]
Biomass fly ash	Cu^2+^ Cd^2+^ Pb^2+^	-	10–250	5.2	0.0025	360	24	42.9 117 534.3	[33]
Fly ash	Cu^2+^ Zn^2+^ Cd^2+^ Pb^2+^	270	0–250 0–260 0–600 0–1000	5.6	0.004	180	22	20.9 7.9 26.9 88.3	[191]
Municipal solid waste incineration fly ash	Cu^2+^	258	40–200	3	-	-	-	84.65	[85]
Fly ash	Pb^2+^	-	10–1000	5.0	-	-	-	497.01	[97]
Industrial waste lithium-silicon-powder	Cu^2+^	-	230	5.0	0.002	2 h	Room temperature	62.3	[60]
Analytical-grade reagents (reference)	Pb^2+^ Cd^2+^ Cu^2+^ Zn^2+^	-	5–1000	3–6 (natural)	0.001	240	Ambient temperature	649 210 90 88	[190]
Coal fly ash	Zn^2+^	-	100 ppm	5	2.5 × 10−3	120	25	35.18 NaP-PEG 36.55 NaP-raw 38.71 NaP-AMP 39.96 NaP-CyOH	[189]
Analytical-grade reagents (reference)	Ca^2+^	-	100–150	7	0.002	15	27	170	[38]
Analytical-grade reagents (reference)	Ca^2+^	-	800	8 12.5	0.002	70	20	92.35 105	[192,193]
Analytical-grade reagents (reference)	Ca^2+^ Zn^2+^	-	100–800	~3	0.004	24 h	Room temperature	68.03 106.38	[148]
Analytical-grade reagents (reference)	Mn^2+^		-	8	1 × 10^−3^	30	Room temperature	44.05	[84]
Analytical-grade reagents (reference)	Fe^3+^	-	100–200	7	1 × 10^−3^	20	27	150	[35]
Natural bentonite	NH^4+^	387	100	6	2 × 10^−4^	360	30	155	[12]
Natural Clinoptilolite (Cpt) Zeolite	Hg^2+^	-	20	7	3.3 × 10^−3^	8 h	25	103.2	[59]
Coal fly ash	Cr^3+^	-	500	-	0.005	24 h	25	83.2	[163]
	Cr^3+^		100	4	0.01	6 h	22	43.6	
	Cu^2+^		100	5	0.01	6 h	22	50.5	
	Cd^2+^ Zn^2+^		100 100	6 6	0.01 0.01	1 h 1 h	22 22	50.8 32.6	
	Ni^2+^		100	6	0.01	1 h	22	50.8	

**Table 19:** Anion/nutrient adsorption capacities and experimental conditions reported for Zeolite-P/GIS materials. Single-solute and real-water studies are not assumed to be equivalent.

Solid Waste Type	Adsorbates (Anions)	CEC (meq/100 g)	Initial Concentration (mg/L)	pH	S/L Ratios (g/mL)	Time (min)	Temperature (°C)	Adsorption Capacity, q (mg/g)	References
								Efficiency (%)	
Coal fly ash	Cl^−^	290	12.4	3.3	0.01	30	-	-	[168]
								48.38	
	F^−^	290	1.2	3.3	0.01	30	-	-	
								75	
	NO^3−^	290	3.8	3.3	0.01	30	-	-	
								52.63	
Coal fly ash	PO_4_^3−^	-	25,000	5.24	0.03	30	-	-	[189]
								91	
	PO_4_^3−^	188	15,000	-	0.01	24 h	-	57.14	[190]
								-	
Coal fly ash	SO_4_^2−^	290	406	3.3	0.01	30	-	-	[176]
								84.97	
Natural bentonite	PO_4_^3−^	387	100	6	0.0002	600	Room Temperature	170	[12]

##### Removal of Organic Contaminant

Zeolite-P and Zeolite-P-based composites have also been evaluated for dyes and other organic contaminants. Because many organic molecules are larger than the intrinsic GIS windows, high uptake can involve external surfaces, secondary mesoporosity, carbon/TiO_2_ or layered-double-oxide composite phases, and surfactant-modified surfaces in addition to the zeolite framework itself. Adsorption values should therefore not be attributed automatically to intracrystalline GIS pore filling.

Moreover, Barghi and coworkers [34] tested the photocatalytic performance of a cost-effective NaP-based nanocomposite (NaP Zeolite@TiO_2_) for the simultaneous removal of methylene blue dye and chemical oxygen demand (COD) under both solar and ultraviolet light. Their results demonstrated that the nanocomposite catalyst achieved over 90% COD removal and approximately 99.6% discoloration of methylene blue at room temperature. The optimal conditions included a TiO_2_ loading of 0.36 g, a catalyst dosage of 0.1 g, 2 h of reaction time, an initial dye concentration of 100 mg/L, and a solution pH of around 7 under solar light irradiation.

On the other hand, the Na-P1-based composite (Na-P1–activated carbon) exhibited a maximum adsorption capacity of 54.13 mg/g for chloroform and 28.16 mg/g for dimethyl disulfide [194].

Furthermore, surfactant-modified Zeolite-P, particularly those modified with hexadecyltrimethylammonium (HDTMA), showed remarkable improvements in their sorption efficiency for organic pollutants. For instance, HDTMA-modified Zeolite Na-P1 exhibited adsorption capacities of 37.7 mg/g for phenol, 52.4 mg/g for p-chlorophenol, and 90.9 mg/g for bisphenol A [195]. Surface modifications using HDTMA create a hydrophobic tail and a hydrophilic head, significantly enhancing the Zeolite-P capacity to capture organic molecules from aqueous solutions.

#### 10.4.2. Regeneration, Reuse, and Matrix Effects

Regeneration is essential for judging whether a high-capacity Zeolite-P adsorbent is practically reusable. Chen et al. [190] reported that a P-type zeolite retained approximately 97% Pb-removal efficiency after five regeneration cycles, demonstrating that ion-exchange sites can be restored under appropriate conditions. By contrast, regeneration performance is contaminant- and matrix-dependent; strong complexation, precipitation, fouling, or structural alteration can progressively reduce recoverable capacity. Accordingly, cycle number, desorbent chemistry, capacity retention, crystal structure after reuse, and the composition of the regeneration effluent should all be reported.

Matrix effects are equally important. Abukhadra et al. [12] tested a bentonite/Zeolite-P composite in groundwater and sewage rather than only in single-solute solutions, illustrating the value of real-matrix evidence. Boudia et al. [98] reported simulated-wastewater dye removal together with multi-cycle reuse and batch-to-column transferability for a Zeolite-P1-based composite. These studies are more process-relevant than single-solute equilibrium tests because competing ions, dissolved organic matter, ionic strength, and pH can change both capacity and selectivity.

The present literature nevertheless remains dominated by model solutions. We therefore distinguish three evidence levels when interpreting performance: (i) single-solute batch tests for intrinsic affinity/mechanism; (ii) multicomponent or real/simulated wastewater tests for matrix tolerance; and (iii) continuous breakthrough plus regeneration for process translation. A material should not be described as industrially validated unless evidence extends beyond the first level.

#### 10.4.3. Adsorption Mechanisms of GIS-Type Zeolite-P

**Figure 17:**
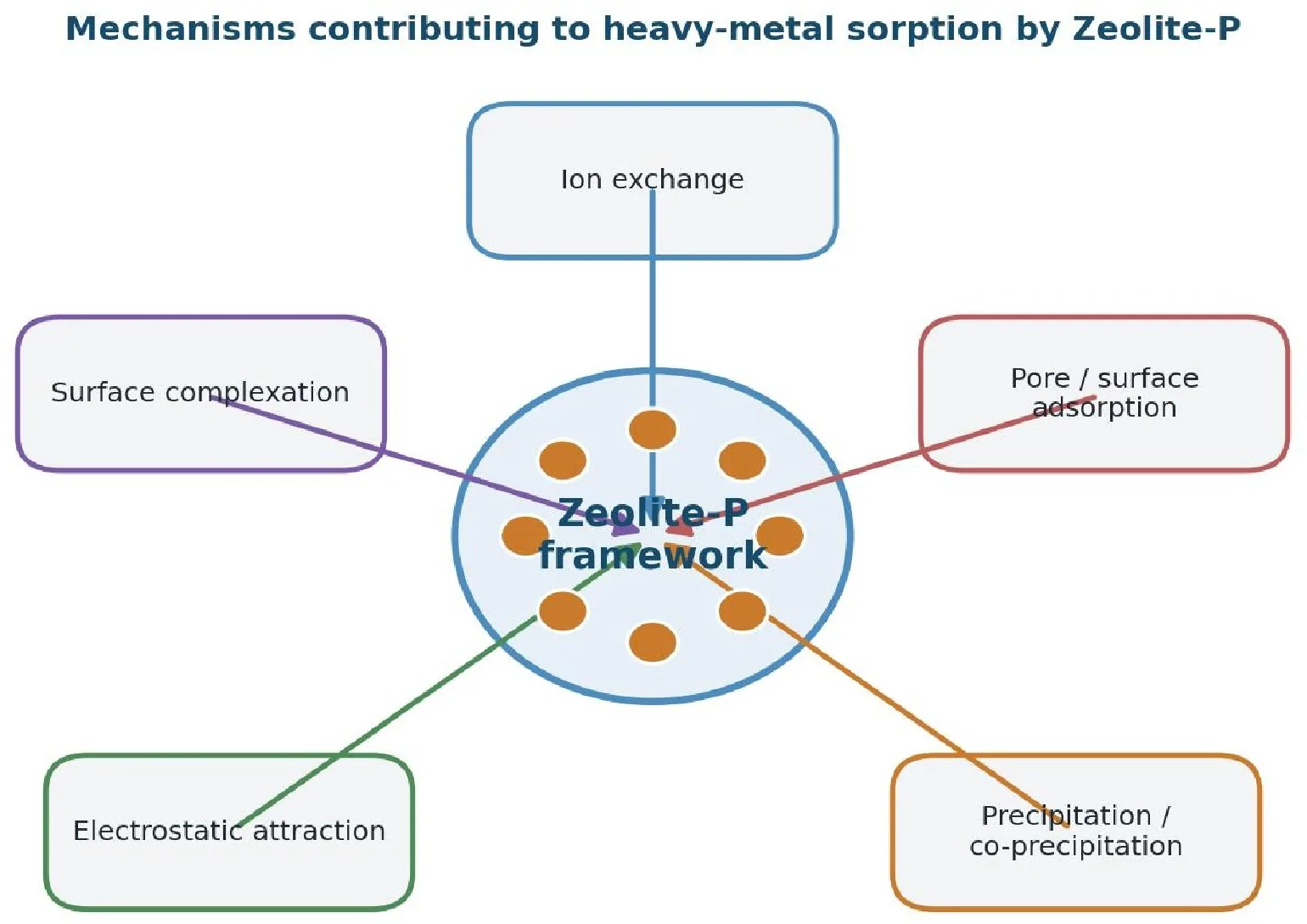
Original schematic summary of mechanisms that can contribute to heavy-metal sorption by Zeolite-P, including ion exchange, surface complexation, electrostatic attraction, precipitation/co-precipitation, and pore/surface adsorption, based on the mechanistic literature discussed in Section 10.4.3.

**Figure 18:**
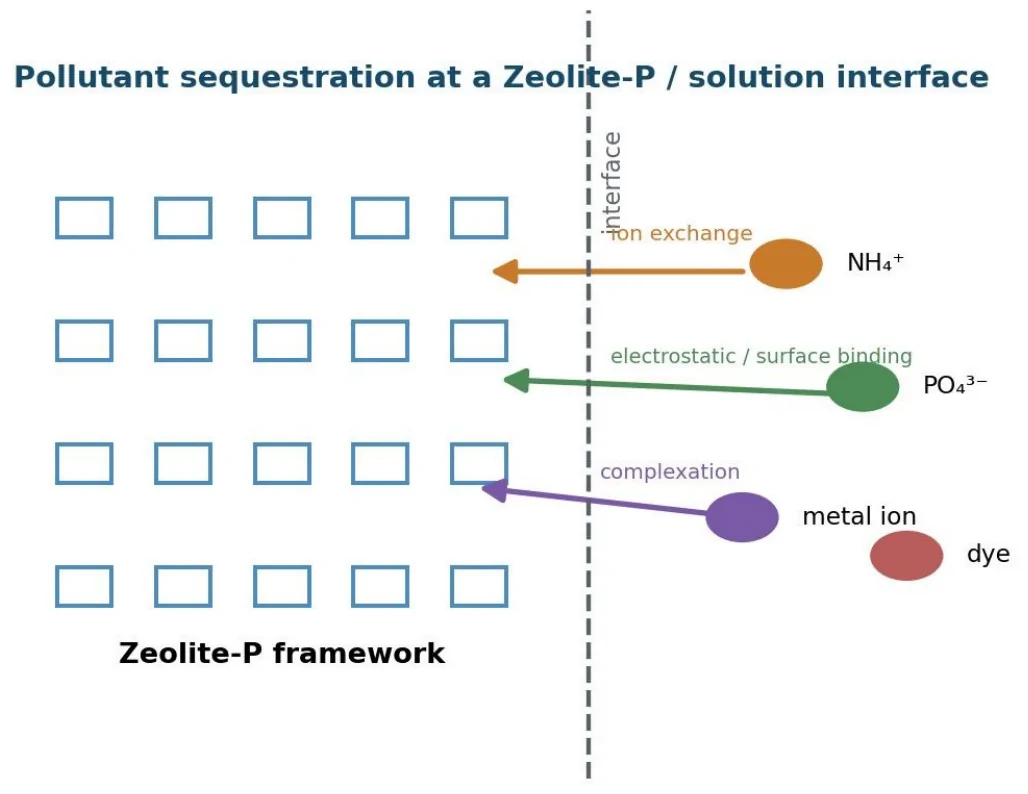
Original schematic of pollutant sequestration at a Zeolite-P/solution interface, illustrating ion exchange, electrostatic/surface binding, and complexation pathways for representative ionic pollutants. The scientific concepts are based on Abukhadra et al. [12] and the literature discussed in Section 10.4.3; no source artwork is reproduced.

For metal cations, the dominant GIS-specific mechanism is exchange of Na^+^/other extra-framework cations, modulated by hydration energy, ionic radius, valence, and access to the small windows; external-surface complexation and, at suitable pH, precipitation/co-precipitation may also contribute. For dyes and other organics, electrostatic attraction, hydrogen bonding, van der Waals interactions, surfactant/composite interfaces, and secondary porosity can dominate because many molecules are too large to enter intrinsic GIS micropores. Gas adsorption is controlled by molecular size, framework/cation electrostatics, and diffusion barriers. These mechanisms should be assigned from complementary evidence rather than from isotherm fitting alone. (Figure 17). (Figure 18). (Table 20).

**Table 20:** Organic-contaminant adsorption capacities and experimental conditions reported for Zeolite-P/GIS materials and composites. Large-molecule uptake may involve external surfaces, secondary porosity, or composite phases in addition to GIS micropores.

Solid Waste Type	Adsorbates (Dyes and Other Organic Compounds)	CEC (meq/100 g)	Initial Concentration (mg/L)	pH	S/L Ratios (g/mL)	Time (min)	Temperature (°C)	Adsorption Capacity q (mg/g)	Ref.
From mordenite(Mor)	Methylene Blue	-	-	6.2	-	72 h	30	15.99	[196]
Coal fly ash	Safranine	-	-	6.34	10	1 d	45	7.015	[193]
Coal fly ash	Thionine			6.39				9.702	
Coal fly ash	Rhodamine B	-	-	6	2.5	60	25	15.92 (NaP-fumed SiO_2_ static) 17.69 (NaP-fumed SiO_2_ (dynamic)	[37]
Coal gasification fine slag	Crystal violet	-	1000	11	4	120	30	222.22	[138]
Natural Bentonite	Methylene Blue Congo Red	387	20	5	0.2	720	Room Temperature	34.85 43.6	[32]
LDH by-products and glass wastes	Methylene Blue Congo Red	-	20	9 5	0.2	210		54.1 62.2	[98]
Illito-Kaolinitic clay	Chloroform Dimethyl disulfide	279	-	-	-	-	-	54.13 28.16	[194]
Fly ash	Phenol p-chlorophenol Aniline Nitrobenzene Bisphenol A Naphthalene	-	50 50 10 1000 100 20	10.3	5	24 h	25	37.7 52.4 3.3 82.3 90.9 4249.3	[195]

#### 10.4.4. Computational Chemistry for Zeolite-P

The most informative computational question for GIS is not whether an ideal framework can adsorb a molecule, but why idealized predictions diverge from experiments on realistic aluminosilicate P1/P2. Early screening of pure-silica GIS predicted strong CO_2_ adsorption and CO_2_/N_2_ selectivity [182,183], whereas experiments on Na-P1/P2 showed much lower light-gas uptake and much stronger water affinity [7].

This discrepancy can be rationalized by model chemistry. Replacing Si with Al introduces framework charge and requires extra-framework cations; those cations alter electrostatic adsorption energies, occupy window/cage volume, interact strongly with water, and can create kinetic gating. A calculation on pure-silica GIS therefore cannot be treated as a quantitative prediction for hydrated Na-P1 or Na-P2. Realistic simulations should specify Si/Al distribution, cation identity/location, hydration state, framework flexibility, and loading. (Table 21).

Grand canonical Monte Carlo (GCMC) is appropriate for equilibrium adsorption and mixture selectivity, density-functional theory (DFT) for local binding geometries and energetics, and molecular dynamics (MD) for diffusion barriers and transport. For GIS, an effective workflow is to validate realistic Na-P1/P2 structural models against XRD/NMR, compute cation- and water-dependent adsorption sites, predict multicomponent isotherms, and then compare those predictions with measured equilibrium and breakthrough data rather than with single-component uptake alone [197,198].

Liquid-phase modeling is a larger gap. Heavy-metal uptake is often interpreted as ion exchange, yet periodic DFT/free-energy calculations that include hydrated ions and explicit water are scarce for GIS. Likewise, dye adsorption should account for molecule size, external surfaces, secondary porosity, and composite phases rather than forcing large molecules into ideal micropores. Practical future strategies include explicit-solvent DFT/MD for ion dehydration and exchange, enhanced-sampling free-energy calculations for window crossing, and multiscale coupling of adsorption/diffusion parameters to column models.

**Figure 19:**
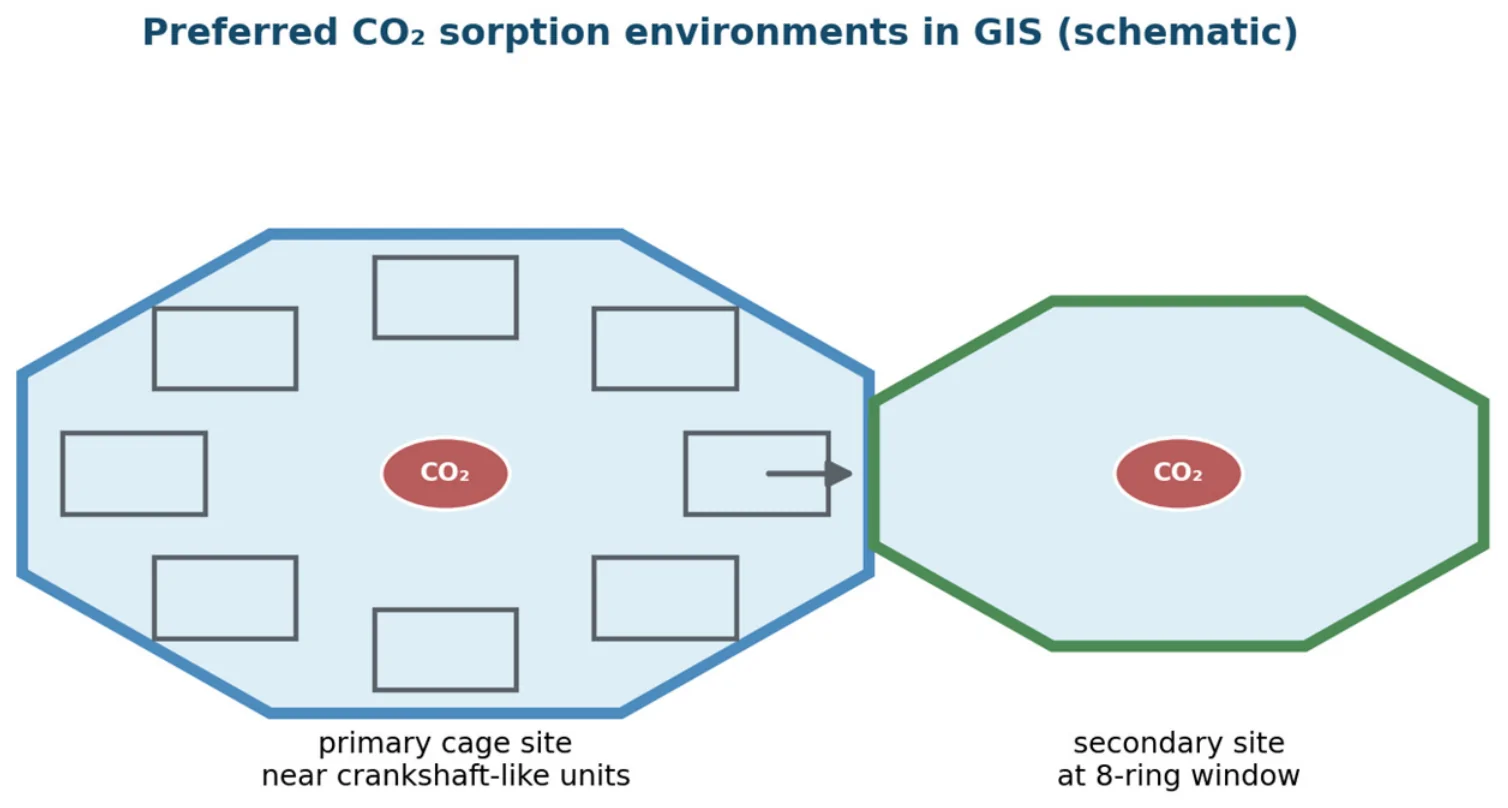
Original schematic of preferred CO_2_ sorption environments in the GIS framework, showing a primary cage site near crankshaft-like units and a secondary site at an 8-ring window, based on the computational findings of Fischer and Bell [182] and structural descriptions by Lozinska et al. [199]; no source structural rendering is reproduced.

The value of computation is therefore strongest when it explains a measured difference—P1 versus P2, Na^+^ versus exchanged cations, dry versus hydrated framework, nano- versus micron-sized crystals—or predicts an experimentally testable trend. Generic descriptions of DFT, MD, or Monte Carlo that are not tied to GIS experiments have been removed from this revision. (Figure 19).

#### 10.4.5. Laboratory-Scale Breakthrough Experiments

Breakthrough experiments provide the clearest bridge from equilibrium adsorption to process behavior because they test a flowing multicomponent stream and capture capacity, mass-transfer kinetics, bed utilization, and competitive displacement simultaneously. For GIS, the available continuous-flow evidence is substantially richer for gases than for aqueous heavy metals or dyes.

**Figure 20:**
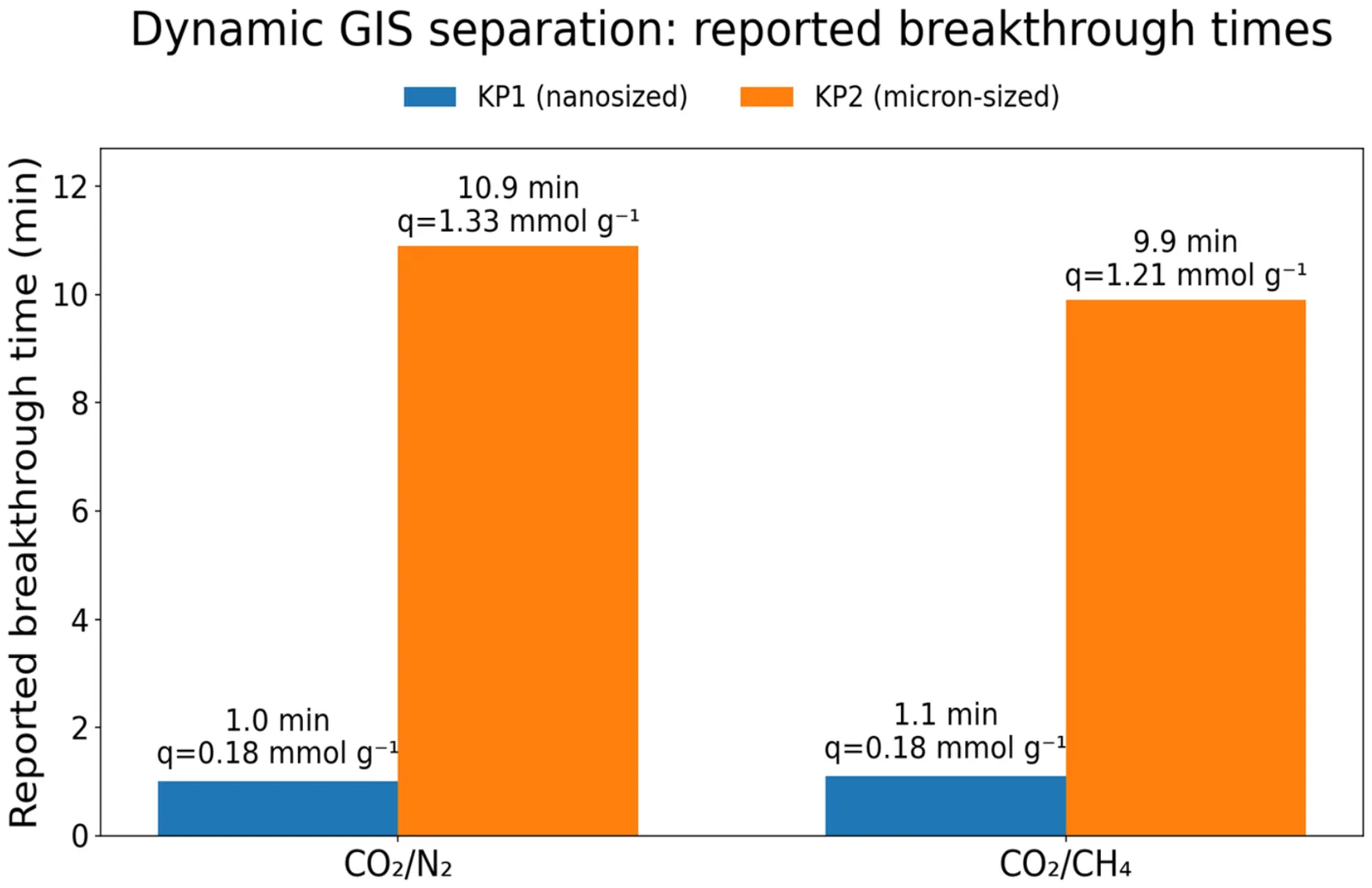
Quantitative comparison of reported breakthrough times for KP1 and KP2 GIS materials in CO_2_/N_2_ and CO_2_/CH_4_ mixtures, with the corresponding reported CO_2_ capacities annotated [186]. Direct comparison with other studies requires matched bed mass, flow rate, pressure, temperature, activation, and mixture composition.

Al Atrach et al. [186] reported dynamic CO_2_/N_2_ and CO_2_/CH_4_ separation for GIS samples with different crystal sizes. In the conditions reported in that study, breakthrough times increased from approximately 1.0 to 10.9 min for CO_2_/N_2_ and from 1.1 to 9.9 min for CO_2_/CH_4_, while the corresponding reported CO_2_ capacities increased from ~0.18 to 1.33 and ~0.18 to 1.21 mmol g^−1^. Figure 20 now visualizes these literature data rather than presenting a generic conceptual curve.

Deng et al. [185] further reported dynamic CO_2_/C_2_H_2_ separation together with a CO_2_ uptake of 3.5 mmol g^−1^ and 128 cm^3^(STP) cm^−3^ at 195 K. These low-temperature/high-selectivity results are scientifically important but should not be extrapolated directly to flue-gas or ambient-temperature operation without matched pressure, temperature, composition, and cyclic stability data.

For aqueous Zeolite-P, fixed-bed evidence remains limited compared with the large number of batch studies. Future column work should report bed dimensions and mass, particle size/shaping, inlet concentration and matrix, flow/residence time, breakthrough criterion, dynamic capacity, pressure drop, regeneration protocol, and performance over repeated cycles.

Empirical models such as Thomas, Yoon–Nelson, Adams–Bohart, and Clark can summarize individual breakthrough curves, but model fit should not be interpreted automatically as proof of a unique rate-limiting mechanism. Mechanistic conclusions require independent transport/kinetic evidence and validation across flow rates, bed depths, and concentrations.

The key translation gap is therefore not another single-solute equilibrium isotherm, but validated batch-to-column performance in multicomponent or real matrices together with regeneration and shaped-adsorbent stability.

## 11. Prioritized Research Needs for GIS-Type Zeolite-P

Fundamental priorities are: (1) resolve P1/P2 phase purity and cation distributions with full-pattern crystallography plus local probes; (2) separate intrinsic GIS microporosity from secondary meso-/macroporosity; (3) quantify hydrated-ion and molecule transport through realistic Al/Na-containing frameworks; and (4) connect DFT/GCMC/MD predictions directly to equilibrium and dynamic experiments. For waste-derived samples, impurity partitioning and leaching under synthesis, use, and regeneration conditions should be treated as part of the material mechanism rather than as an afterthought.

A further fundamental priority is (5) to quantify Brønsted, Lewis and basic site densities of waste-derived and reagent-derived GIS side by side, at matched Si/Al ratio, phase purity, cation form and activation, using probes that can enter the 8-ring channels (NH_3_, CO, CO_2_) together with ^1^H and ^27^Al MAS NMR, so that the effect of waste origin can be separated from the general physicochemical properties of the zeolite. As a minimum reporting set for waste-derived GIS, we recommend: precursor composition; bulk and framework Si/Al; phase purity and crystallinity from full-pattern analysis; cation form and inventory; N_2_ and CO_2_ porosimetry with the probe stated; acid/base-site data with the probe stated; leaching results; and complete test conditions (concentration, solid-to-liquid ratio, pH, temperature, time, matrix).

Applied and engineering priorities are: (1) test multicomponent and real wastewater rather than only single-solute model solutions; (2) report regeneration chemistry, capacity retention, secondary waste, and structural stability over sufficient cycles; (3) compare powder and shaped bodies with binder fraction, strength, pressure drop, diffusion, and capacity; (4) perform breakthrough tests under realistic bed and flow conditions; and (5) evaluate cost and life-cycle impact using a functional unit tied to delivered treatment (for example, pollutant removed over adsorbent lifetime). These priorities distinguish material discovery from process validation and provide a clearer path toward industrial deployment.

## 12. Summary

Waste-derived Zeolite-P is best understood as a family of GIS-type microporous adsorbents whose performance depends on polymorph (P1/P2), Si/Al ratio, cation form, phase purity, crystal size, and secondary textural porosity. This review shows that mesoporosity observed in some samples is generally associated with aggregation, interparticle voids, defects, or hierarchical architectures rather than with the intrinsic GIS framework. Waste precursors can provide useful Si/Al sources, but their economic and environmental advantages are route-specific and must include pretreatment, alkali/water use, energy, washing, effluent treatment, shaping, and leaching safety.

For applications, the strongest evidence supports ion exchange, water purification, dehydration, and selected gas-separation uses. Idealized pure-silica simulations can overpredict CO_2_ performance relative to realistic aluminosilicate Na-P1/P2, emphasizing the need to connect computation with measured cation occupancy, hydration, diffusion, and breakthrough behavior. The next advances should move beyond high single-solute batch capacities toward phase-resolved, multicomponent/real-water, regenerable, shaped, and continuously operated GIS adsorbents evaluated with process-level techno-economic and life-cycle metrics.

## Figures and Tables

**Table 1:** Representative XRD reflections reported for GIS P1/P2 materials. Values are approximate and composition/hydration dependent; the table is intended for pattern guidance, not single-peak polymorph assignment.

Polymorphs	Relative Miller Index (hkl)	Relative Angle (°2θ)	Plausible Interval (°2θ)
P1	(110) *	~12.3–12.5	[12.2–12.6]
P2		~12.5–12.7	[12.4–12.8]
P1	(200) *	~17.7–17.8	[17.6–17.9]
P2		~17.8–18.0	[17.7–18.1]
P1	(211)	~21.7–21.8	[21.6–21.9]
P2		~21.9–22.0	[21.8–22.1]
P1	(220)	~26.2–26.3	[26.1–26.4]
P2		~26.3–26.5	[26.2–26.6]
P1	(321)	~27.1–27.2	[27.0–27.3]
P2		~27.3–27.4	[27.2–27.5]
P1	(221)	~30.0–30.1	[29.9–30.2]
P2		~30.1–30.3	[30.0–30.4]
P1	(321)	~34.2–34.3	[34.1–34.4]
P2		~34.3–34.5	[34.2–34.6]

* Representative reflections. Exact 2θ values depend on composition, hydration, instrumental calibration, and radiation source (Cu Kα assumed here, λ = 1.5406 Å).

**Table 2:** Comparative gas adsorption capacities as a function of the distinct morphologies of GIS-type Zeolite forms (Na-P1 and Na-P2).

Morphology	Framework Description	H_2_O (mmol/g)	CO_2_ (mmol/g)	N_2_ (mmol/g)	Ref.
Na-P1: pseudo-cubic/polycrystalline morphology (see Figure 5)	GIS-P1; 8-membered-ring channel framework (see Figure 3)	10.76	0.027	0.005	[7]
Na-P2: more angular/transformed morphology (see Figure 5)	GIS-P2; 8-membered-ring channel framework (see Figure 3)	7.20	0.040	0.008	[6]

**Table 3:** Characteristic FTIR bands of GIS-type zeolites, adapted from [15].

**Internal Tetrahedral Linkages**	**Assignment (cm^−1^)**
T-O bend	4000–420
Bridging OH groups in Al–OH-Si	
Symmetric stretch	1250–950
Symmetrical stretch vibrations of bridge ties—νs Si–O-Si; symmetrical stretch vibrations of bridge ties—νs Si–O-Al	
Asymmetric stretch	
Asymmetric stretch vibrations of bridge ties—νas Si–O and νas Si–O(Al)	720–650
**External Linkages**	**Assignment (cm^−1^)**
Asymmetric stretch	1150–1050 shoulder
Symmetric stretch	820–750
Double ring	650–500
(Complex band) symmetric stretching vibrations νs of bridging bonds Si–O-Si and bending vibrations—δ O-Si–O	555
Bending vibrations—δ O-Si–O occurring “antiphase”	468
Pore opening	420–300
Bending vibrations—δ O-Si–O and δ O–Al-O	377

**Table 4:** Chemical composition by XRF analysis of synthesized Zeolite NaP from Cpt.

Composition	SiO_2_	Al_2_O_3_	CaO	Fe_2_O_3_	MgO	TiO_2_	K_2_O	Na_2_O	P_2_O_5_	BaO	SrO	Cr_2_O_3_	MnO	LOI
**Mass (%)**	43.66	15.49	4.10	3.77	1.18	0.56	2.76	11.28	0.04	0.12	0.02	<0.01	0.05	17.24

LOI: Loss On Ignition.

**Table 5:** Results of ICP-OES analysis of supernatant used in hydrothermal synthesis of Zeolite-P from Volclay [14].

Element	Concentration (mg/L)	Concentration (mmol/L)	RSD (%)
Na	4469	2914.56	1.4
Si	1536	822.86	0.2
Al	83.91	46.62	1.0
Fe	12.41	3.32	1.1
K	123.5	47.31	0.7
Mg	4.185	2.58	2.0

**Table 6:** Summary of textural properties of P-type Zeolites synthesized from various industrial waste-derived raw materials, including surface area, pore characteristics, and IUPAC isotherm types.

Raw Materials	Surface Area (m^2^ g^−1^)		Pore Volume (cm^3^ g^−1^)		Average Pore Size (nm)	IUPAC Isotherm Type	Ref.
	BET Surface Area SBET	Micropore Surface Area Smicro-	Mesopore Volume Vmesopore	Micropore Volume Vmicropore			
Coal fly ash	26.8	21.0	0.02107	0.03476	5.48	IV	[28]
Coal fly ash	22.3	-	0.100	0.0026	13.62	-	[29]
Analytical-grade reagents (reference)	49	-	3.7	-	20.0 ± 3.0	I	[30]
Coal fly ash	68.81	8.99	-	0.132	1.092	-	[31]
bentonite	123	-	0.01	0.36	1.2	S-type	[32]
Agro-Waste	129	64	0.18 (total)		-	IV	[33]
Biomass fly ash	65.0	-	-	0.0074	1.1	IV	[33]
Analytical-grade reagents (reference)	46.00	44.00	0.03	0.20	1.35	I and IV	[34]
Analytical-grade reagents (reference)	13.0	6.9	0.016	0.0037	19.1	IV	[35]
Coal fly ash	47.93	8.51	0.118	0.00315	9.8	II and IV	[36]
Coal fly ash	26.8	20.95	0.015	0.00880	3.856	IV	[37]
Analytical-grade reagents (reference)	6.2	5.3	0.009	0.0029	19.0	IV	[38]
Analytical-grade reagents (reference)	29.19	21.36	0.08	0.03	18.61	IV	[23]
	41.37	29.45	0.14	0.06	33.06		
	36.93	29.35	0.14	0.07	44.92		
	42.66	33.52	0.17	0.07	38.48		
	45.11	32.39	0.18	0.07	44.92		
	43.48	33.79	0.16	0.13	44.92		
	46.50	36.22	0.18	0.08	44.92		

Type IV/H3 or H4 hysteresis, when reported, is interpreted here as secondary textural mesoporosity; it is not evidence that the GIS framework itself is mesoporous.

**Table 7:** Overview of hydrothermal method and key parameters for Zeolite-P synthesis from various industrial waste precursors.

Raw Materials	Reaction Condition	SiO_2_/Al_2_O_3_	References
Paper sludge incineration ash	130 °C for 5 h	-	[29,30,36,37,38,46,47,48,49,50,51,52,53,54,55,56,57,58,59,60,61,62,63,64,65,66]
Paper sludge with addition of diatomite	90 °C for 12 h	1.57	
Kaolin/metakaolin	180 °C for 3 days (SiO_2_/Na_2_O = 5.38)	7.0	
Coal fly ashes	140 °C for 48 h	2.78	
Sodium aluminate (Al_2_O_3_ 50–56 wt.%, Na_2_O 40–45 wt.%), Sodium silicate (SiO_2_ 25 wt.%, Na_2_O 8.06 wt.%)	100 °C for 48 h (Na_2_O:SiO_2_ = 2.1:3.8)	3.8	
Fumed SiO_2_ (0.014 μm, 200 ± 25 m^2^ g^−1^, Sigma), and NaAlO_2_ (anhydrous, Riedel-de Haen, technical)	90 °C for 3 d (Na_2_O:SiO_2_ = 5.5:4)	4	
Rice husk ash	100 °C for 48 h (SiO_2_/Na_2_O = 1.81)	3.8	
Sodium aluminate and Sodium silicate	100 °C for 48h (Na_2_O:SiO_2_ = 2.1:3.8)	3.8	
Sigma-Aldrich reagents: Silica fume (99.8%, m.p. 1700 °C), Sodium aluminate (NaAlO_2_, 50–56 wt.%)	100 °C for 4 days (Na_2_O:SiO_2_ = 2.76: 2.5)	1.40	
Fly ash-based geopolymer	100 °C for 24 h	1.4	
Natural Clinoptilolite without any chemical pre-treatment	90 °C for 5 h and 72 h	5.44	
Hazardous aluminum solid waste	120 °C for 6 h	2.0	
Coal fly ash microspheres	80 °C for 26 h, atmospheric pressure	1.08	
Waste pearlite	100 °C for 24 h	4.2	
Fly ash	120 °C for 8 h (Na_2_O:SiO_2_ = 1.66:1)	1.96	
Coal fly ash	120 C for 8 h	2.46	
Clinoptilolite with pre-treatment	80 °C for 24 h under atmospheric pressure	-	
Lithium-silicon-powder	60–140 °C for 1–20 h	-	
1-Dodecyl-3-methylimidazolium chloride [C12mim][Cl] Imidazolium-based ionic liquids	87 °C for 54, 78 and 102 h (SiO_2_/Na_2_O = 1.5)	15.43	
Coal fly ash	120 °C for 12 h (Na_2_O:SiO_2_ = 0.70)	2.37	
Kaolinitic rock	65 °C for 24 h and 100 °C for 8 h (SiO_2_/Na_2_O = 1.74)	6.25	
Clinoptilolite/mordenite-rich Zeolite	96 °C for 60 h “top-down approach”	1.75	
Natural clinoptilolite Zeolite	100 °C for 4days (Na_2_O:SiO_2_ = 8.40:56.78)	2.4	
Aluminum isopropoxide (98%) (Al(O-i-pr)), aluminum hydroxide hydrate (Al(OH)_3_), and sodium aluminate anhydrous (NaAlO_2_), Ludox AS-40 colloidial silica, and sodium silicate solution (reagent grade)	100 °C for 48 h (Na_2_O:SiO_2_ = 10:20)	10	
Waste glass and aluminum	60 °C for 6 days	-	
Fly ash waste	140 °C for 50 h	-	

**Table 8:** Summary of alkali fusion–hydrothermal methods and key parameters for Zeolite-P synthesis from industrial waste precursors.

Raw Materials (RM)	Reaction Condition (Alkali Fusion; Hydrothermal Treatment)	RM:NaOH	References
Fly ash	1 h at 550 °C;	1:1.2	[14,33,69,70,71]
	120 °C for 4 h		
Kaolin/Metakaolin	3 h at 800 °C;	29.49:3.11	
	100 °C for 72 h		
Red mud	60 min at 650 °C;	-	
	120 °C for 7 h		
Biomass fly ash	3 h at 600 °C;	1:2	
	120 °C for 24 h		
Clay fraction > 2 µm	2.5 h at 750 °C;	1:1.2	
	150 °C for 18 h		

**Table 9:** Summary of sol–gel method and key parameters for Zeolite-P synthesis from industrial waste precursors.

Gel Composition (Molar Basis)	Crystallization Time	Crystallization Temperature (°C)	Main Product	References
Na_8_Al_8_Si_8_O_32_·15.2H_2_O	5 days	100	Na-P2	[72,73,74,75,76,77]
2.2SiO_2_:Al_2_O_3_:5.28NaF:105.6H_2_O	60 days	85	Na-P1	
SiO_2_:0.25Al_2_O_3_:3Na_2_O:410H_2_O	3 h	110	Na-P1	
3.75SiO_2_:0.2Al_2_O_3_:4.3Na_2_O:220H_2_O	42 h	100	Na-P1	
0.0065NaAlO_2_:0.023Na_2_Si_3_O_7_ (seed);				
0.04NaAlO_2_:0.147Na_2_Si_3_O_7_ (feed)	24 h	100	Na-P1	
17Na_2_O:Al_2_O_3_:17SiO_2_:345H_2_O (seed);	2 h	150	Na-P2	
3Na_2_O:Al_2_O_3_:8SiO_2_:209H_2_O (overall)				

**Table 10:** Summary of microwave methods and key parameters for Zeolite-P synthesis from low-cost precursors.

Raw Materials	Reaction Condition	SiO_2_/Al_2_O_3_	References
Fly ash	175 °C for 30 min (1000 W)	-	[72,79,80,81,82,83,84]
Alumatrane and silatrane	140 °C for 60 min	-	
Gel of MCM-22	130 °C for 3 h (300W)	2.64	
Perlite	30 °C for 55 min (100 W)	-	
Gel of NaY Zeolite	150 °C for 1 h (900 W)	3.85	
Waste glass cullet	90 °C for 10 min (50–100 W)	8.2	
Amorphous hydrophilic fumed silica—SiO_2_	130 °C for 80 min (1000 W)	2.0	

**Table 11:** Summary of ultrasound method and key parameters for Zeolite-P synthesis from low-cost precursors.

Raw Materials	Reaction Condition	SiO_2_/Al_2_O_3_	References
Ludox AS-40 + Al tri-sec-butoxide	150 W for 3 h irradiation (Room temperature)	5	[31,55,81,86,87]
Perlite	100 W for 55 min irradiation (30 °C)	-	
Coal fly ash	125 W for 30 min irradiation (100 °C)	2.13	
Natural clinoptilolite	15 W for 60 min irradiation (90 °C)	2.82	
Coal fly ash	1200 W for 4 h irradiation (90 °C)	4	

**Table 12:** Practical comparison of process considerations for major Zeolite-P synthesis routes, based on the literature discussed in Section 4.

Synthesis Methods	Key Process Considerations
Conventional hydrothermal synthesis	Comparable in simplicity to synthesizing Zeolite-P from pure materials, with lower energy consumption; hydrothermal crystallization demands long reaction times, often resulting in lower yield and purity of the synthesized Zeolite-P, more waste, low conversion.
Alkali fusion-hydrothermal synthesis	Enables efficient activation of tailings, faster speed of reaction rates yielding Zeolite-P with high conversion rates and purity, and less waste; the high temperatures required for fusion significantly increase process costs, relatively expensive.
Sol–Gel	Enables control over stoichiometry and composition of Zeolite-P, possibility to predetermine the structure of Zeolite-P, ease of processing; susceptible to shrinkage and cracking during drying; high precursor cost; moisture sensitivity; challenges in ensuring chemical homogeneity.
Ultrasonic hydrothermal synthesis	Reduces synthesis time, enhances physicochemical properties of the products, and lowers energy consumption; only promotes dissolution of the amorphous(AM) phase in solid waste, with limited effect on the crystalline phase; the mechanism of ultrasonically assisted crystallization remains unclear.
Microwave-assisted hydrothermal synthesis	Microwave heating offers faster and more uniform heating than conventional methods, effectively improving tailing solubility and the P zeolitization process; large-scale industrial applications are still lacking.
Solvent-free synthesis	Environmentally friendly; zero liquid discharge, reduces energy and water consumption; often requires long crystallization times; product quality depends strongly on precursor homogeneity.

**Table 13:** Illustrative material and equipment inputs relevant to Zeolite-P synthesis. Values are screening-level inputs only; they are not normalized across studies, years, scales, or geographic electricity/reagent prices, and waste-derived precursor cost must include collection, transport, pretreatment, and residue management.

Material	Used Quantity (g or mL)	Purchasing Cost (USD/g)	Cost of Used Quantity (USD)	
Waste-derived precursors	-	Site-specific	Include collection, transport, drying/milling, washing/pretreatment, and residue handling	
Deionized water	-	0.001	-	
NaOH pellets	-	0.6	-	
Equipment	Time (h)	Max. power (kW)	Unit cost of power	Energy cost (USD/h)
Hot plate	-	1.0	0.16	-
Dry	-	1.0		-
Crushing (ball mill)	-	0.5		0.08
Centrifuge	-	0.25		0.04
Calcination (muffle furnace)	-	3.0		0.48
Hydrothermal autoclave	-	1.5		0.24
Microwave reactor	-	2.0		0.32
Ultrasonic bath	-	0.15		0.024
Probe sonicator	-	0.5		0.08

Note: Robust techno-economic and life-cycle comparison requires a common functional unit (for example, 1 kg phase-pure shaped adsorbent or 1 kg pollutant removed over repeated cycles), defined system boundaries, product yield/purity, shaping, regeneration, and wastewater-treatment burdens.

**Table 15:** Chemical composition of common industrial wastes used as low-cost raw materials for Zeolite-P synthesis.

Solid Waste Type	SiO_2_	Al_2_O_3_	CaO	MgO	Fe_2_O_3_	K_2_O	Na_2_O	TiO_2_	References
Coal fly ash	53.02	22.74	5.86	1.82	5.75	2.07	0.10	0.94	[112]
Coal gasification slag	53.44	17.21	10.12	1.92	11.23	2.27	1.3	-	[113]
Rice husk ash	68.28	1.28	0.37	3.59	0.54	3.14	3.78	0.07	[114]
Solar panel waste glass	72.33	1.90	8.98	2.62	0.03	0.04	12.87	-	[115]
Sandblasting waste	10.25	45.60	5.47	-	12.23	0.16	1.23	21.10	[115]
Sugarcane bagasse ash	66.28	15.10	3.75	0.51	3.40	5.15	1.55	-	[116]
Fruit waste	55.98	2.71	8.95	1.08	1.36	28.72	-	-	[117]
Blast furnace slag	34.58	14.78	40.1	5.29	-	-	-	-	[118]
Paper sludge ash	35.9	22.8	33.2	4.5	0.9	-	0.6	2.2	[46]
Clay materials (kaolin, smectite)	46.5	41.18	-	0.04	0.19	0.13	0.18	0.13	[70]
Lithium slag	70.67	27.24	0.29	0.16	0.52	0.22	0.13	-	[119]
Waste of iron mine tailings/iron ore tailing	67.58	8.70	5.78	4.37	7.42	2.32	2.15	0.33	[120]
Waste pearlite	75.39	13.36	0.97	0.11	1.22	4.11	4.61	-	[57]
Clinoptilolite	65.87	12.10	2.62	1.23	2.49	3.43	1.73	-	[55]

**Table 16:** Structural and adsorption-performance comparison of GIS/Zeolite-P materials synthesized from different precursors and routes. Phase identity/purity is retained as reported by the cited studies; mesopore diameters are standardized to nm and pore volumes to cm^3^ g^−1^.

Synthesis from Waste	Synthesis Method	XRD Evidence	SEM Morphology	N_2_ Adsorption Evidence	Porous and Textural Properties	Adsorption Capacity	Ref.
Fly ash	Hydrothermal synthesis	Reported in cited study	Reported in cited study	-	-	2.2 meq/g (Ammonium)	[163]
Fly ash	Alkaline fusion-assisted hydrothermal treatment	Reported in cited study	Reported in cited study	Reported in cited study	SBET = 18.03 m^2^/g SMicro = 8.99 m^2^/g V_total = 0.05 cm^3^/g D_avg = 9.98 nm	7.4 meq/g (Ammonium)	[164]
Fly ash	Microwave-assisted method	Reported in cited study	Reported in cited study	Reported in cited study	S_BET = 72.05 m^2^ g^−1^ S_micro = 17.10 m^2^ g^−1^ V_total = 0.134 cm^3^ g^−1^ D_avg = 11.07 nm	0.48 meq/g (Ammonium)	[31,132]
Fly ash	Thermo-sonochemical treatment	Reported in cited study	Reported in cited study	Reported in cited study	S_BET = 68.81 m^2^ g^−1^ S_micro = 8.99 m^2^ g^−1^ V_total = 0.132 cm^3^ g^−1^ D_avg = 10.92 nm	0.43 meq/g (Ammonium)	[31,132]
Fly ash	Solvent-free process	Reported in cited study	Reported in cited study	Reported in cited study	SBET = 18.5 m^2^/g V_totall = 0.0007 cm^3^/g D_avg = 12.49 nm	22.9 mg/g (Ammonium)	[88]
Rice husk	Hydrothermal synthesis	Reported in cited study	Reported in cited study	-	S_BET = 50 m^2^ g^−1^ D_avg = 4.08 nm	19.053 mg/g (Phosphate)	[165]
Paper sludge ash	Hydrothermal synthesis	Reported in cited study	Reported in cited study	Reported in cited study	-	130 cmol/kg (Ammonium)	[47]
Diatomite	Hydrothermal synthesis	Reported in cited study	Reported in cited study	Reported in cited study	SBET = 56–60 m^2^/g	300 cmol/kg (Calcium)	[166]
Blast furnace slag	Hydrothermal synthesis	Reported in cited study	Reported in cited study	-	SBET = 60 m^2^/g	-	[120]
Ladle furnace slag	Hydrothermal synthesis	Reported in cited study		Reported in cited study	S_BET = 27.3 m^2^ g^−1^ S_t-plot = 28.66 m^2^ g^−1^ V_total = 0.050 cm^3^ g^−1^ D_avg = 16.7 nm	-	[158]
Analytical grade reactants	Hydrothermal synthesis	Reported in cited study	Reported in cited study	Reported in cited study	S_BET = 853.6 m^2^ g^−1^ V_total = 0.34 cm^3^ g^−1^ Characteristic micropore width ≈ 0.46–0.51 nm; additional meso/macroporosity is sample-dependent	423.4 cm^3^/g (Water vapor)	[23]

**Table 17:** Acid and base site types in GIS-type Zeolite-P, their origin, how precursor origin and preparation influence them, and appropriate probes.

Detection (GIS-Specific Caveat)	Effect of Precursor Origin and Preparation	Formation in GIS	Site Type
NH_3_-TPD/IR; ^1^H MAS NMR. Pyridine-IR (≈1545 cm^−1^) detects only external sites (pyridine ≈ 5.4 Å > GIS window)	Origin sets Si/Al and hence maximum site number; Al-rich frameworks are acid-labile, so H-form formation causes partial dealumination/amorphization; strength per site is low at high Al density	Forms only after NH_4_^+^ exchange/calcination or hydrolysis of polyvalent cations (Ca^2+^, Mg^2+^, Fe^3+^); negligible in Na-P	Brønsted—bridging Si(OH)Al
IR ν(OH) ≈ 3740 cm^−1^ (terminal silanols); ^1^H MAS NMR; low-temperature NH_3_-TPD peak	Increase with small crystals (microwave/ultrasound routes), low crystallinity and residual glass/gel	External surface, defects, amorphous residue	Weak Brønsted/H-bond donors—silanol, Al–OH
^27^Al MAS NMR (octahedral Al ≈ 0 ppm); IR of probes (pyridine ≈ 1450 cm^−1^, surface only)	Harsher fusion/calcination and acid washing increase extra-framework Al	Generated by dealumination during fusion, ion exchange, acid or thermal treatment	Lewis—extra-framework Al
CO/CO_2_ IR; cation inventory by XRF/ICP-OES; matched dehydration	Waste feeds contribute Ca, Mg, K, Fe that persist if exchange/washing is incomplete; hydration state controls strength	Intrinsic to Na-P; polarizing power increases Na^+^ < Ca^2+^ < Mg^2+^	Lewis—charge-compensating cations (Na^+^, K^+^, Ca^2+^, Mg^2+^)
XRD, XRF, XPS (Fe 2p); characterize the unconverted residue as a blank	Specific to waste origin; depends on fly-ash/slag composition and conversion yield	Not part of the GIS framework	Lewis/redox—impurity phases (Fe/Ti oxides, residual mullite, glass)
CO_2_-TPD/CO_2_-IR; slurry pH after washing; Hammett indicators (surface only)	Al-rich GIS is strongly basic; incompletely washed NaOH/carbonate inflates apparent basicity	Basicity rises with Al content and electropositive cations [110]	Basic—framework O (Si–O–Al), residual alkali

**Table 21:** Representative computational parameters used for periodic GIS unit-cell calculations. Such parameters should be reported together with framework composition, Al distribution, cation positions, hydration state, force field/functional, and adsorbate loading when comparing simulation with experiment.

Plane-Wave Energy Cutoff	k-Point Mesh	Smearing Method	Ionic Relaxation Force Criterion	SCF Convergence Criterion	Lattice Constants		
					Na-P1	Na-P2	Si-P1
680 eV	2 × 2 × 2	Gaussian-smearing, kbT = 0.1 eV	0.01 eV Å^−1^ per atom	10^−8^ eV	a = 9.99 Å b = 9.99 Å c = 9.94 Å	a = 9.79 Å b = 10.00 Å c = 10.02 Å	a = 9.99 Å b = 9.99 Å c = 9.99 Å

## Data Availability

No new data were created or analyzed in this study. Data sharing is not applicable to this review article.
